# Leukocyte dynamics after intracerebral hemorrhage in a living patient reveal rapid adaptations to tissue milieu

**DOI:** 10.1172/jci.insight.145857

**Published:** 2021-03-22

**Authors:** Brittany A. Goods, Michael H. Askenase, Erica Markarian, Hannah E. Beatty, Riley S. Drake, Ira Fleming, Jonathan H. DeLong, Naomi H. Philip, Charles C. Matouk, Issam A. Awad, Mario Zuccarello, Daniel F. Hanley, J. Christopher Love, Alex K. Shalek, Lauren H. Sansing, the ICHseq Investigators

**Affiliations:** 1 Department of Chemistry and Institute for Medical Engineering and Science, Massachusetts Institute of Technology, Cambridge, Massachusetts, USA.; 2 Broad Institute, Harvard University and Massachusetts Institute of Technology, Cambridge, Massachusetts, USA.; 3 Department of Neurology, Yale University School of Medicine, New Haven, Connecticut, USA.; 4 Koch Institute for Integrative Cancer Research, Massachusetts Institute of Technology, Cambridge, Massachusetts, USA.; 5 Department of Immunobiology and; 6 Department of Neurosurgery, Yale University School of Medicine, New Haven, Connecticut, USA.; 7 Department of Neurosurgery, University of Chicago, Chicago, Illinois, USA.; 8 Department of Neurosurgery, University of Cincinnati, Cincinnati, Ohio, USA.; 9 Brain Injury Outcomes Division, Johns Hopkins School of Medicine, Baltimore, Maryland, USA.; 10 Department of Chemical Engineering, Massachusetts Institute of Technology, Cambridge, Massachusetts, USA.; 11 Ragon Institute, Harvard University, Massachusetts Institute of Technology, and Massachusetts General Hospital, Cambridge, Massachusetts, USA.; 12 Division of Health Science and Technology, Harvard Medical School, Boston, Massachusetts, USA.; 13 Program in Computational & Systems Biology, Massachusetts Institute of Technology, Cambridge, Massachusetts, USA.; 14 Department of Immunology, Massachusetts General Hospital, Boston, Massachusetts, USA.; 15 Human and Translational Immunology Program, Yale School of Medicine, New Haven, Connecticut, USA.; 16 The ICHseq Investigators are detailed in Supplemental Acknowledgments.

**Keywords:** Immunology, Neuroscience, Stroke

## Abstract

Intracerebral hemorrhage (ICH) is a devastating form of stroke with a high mortality rate and few treatment options. Discovery of therapeutic interventions has been slow given the challenges associated with studying acute injury in the human brain. Inflammation induced by exposure of brain tissue to blood appears to be a major part of brain tissue injury. Here, we longitudinally profiled blood and cerebral hematoma effluent from a patient enrolled in the Minimally Invasive Surgery with Thrombolysis in Intracerebral Hemorrhage Evacuation trial, offering a rare window into the local and systemic immune responses to acute brain injury. Using single-cell RNA-Seq (scRNA-Seq), this is the first report to our knowledge that characterized the local cellular response during ICH in the brain of a living patient at single-cell resolution. Our analysis revealed shifts in the activation states of myeloid and T cells in the brain over time, suggesting that leukocyte responses are dynamically reshaped by the hematoma microenvironment. Interestingly, the patient had an asymptomatic rebleed that our transcriptional data indicated occurred prior to detection by CT scan. This case highlights the rapid immune dynamics in the brain after ICH and suggests that sensitive methods such as scRNA-Seq would enable greater understanding of complex intracerebral events.

## Graphical abstract

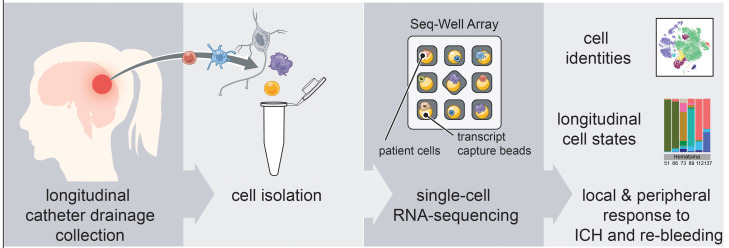

## Introduction

Intracerebral hemorrhage (ICH), brain bleeding often caused by hypertension, has a worldwide incidence of more than 3 million cases per year, limited treatment options, and high mortality rate (40%–60% worldwide; refs. 1, 2). To date, surgical approaches to remove the hemorrhage have not improved functional outcomes (3, 4). The phase III clinical trial of Minimally Invasive Surgery with Thrombolysis in Intracerebral Hemorrhage Evacuation (MISTIE III) was designed to test whether minimally invasive catheter evacuation followed by thrombolysis would improve functional outcome in patients with ICH. The trial’s approach used image-guided minimally invasive surgery to aspirate the liquid component of the hemorrhage followed by placement of a sutured indwelling catheter to liquify and drain remaining hemorrhage over several days with the aid of recombinant tissue plasminogen activator (tPA). The trial found that surgery was safe and reduced mortality, but did not improve overall functional outcomes 365 days after ICH (5). As part of a trial substudy (ICHseq), daily samples of both hematoma effluent from the catheter and peripheral blood were collected. These samples provided a unique opportunity to study the immune response to ICH over time in living patients.

Here, we describe longitudinal cellular characterization of hematoma effluent and blood from a patient who was enrolled in MISTIE III using single-cell RNA-Seq (scRNA-Seq) (6). We define distinct leukocyte phenotypes that may dynamically reflect local immune responses in the brain as well as the emergence of cells resembling peripheral blood leukocytes that we propose identify the onset of repeat tissue exposure to blood (in this case, an asymptomatic rebleeding event or second local exposure to blood) later detected by CT scan. Additionally, we show that leukocyte transcriptional programs in the patient’s blood returned to a baseline comparable to age-matched control blood 2.5 years after ICH. To our knowledge, this case report is the first longitudinal single-cell genomic characterization of cellular infiltrate in acute brain injury in a living patient and, importantly, underscores the utility of single-cell analyses for understanding dynamic immune responses in the brain and other tissues.

## Results

### Patient timeline.

**Figure 1:**
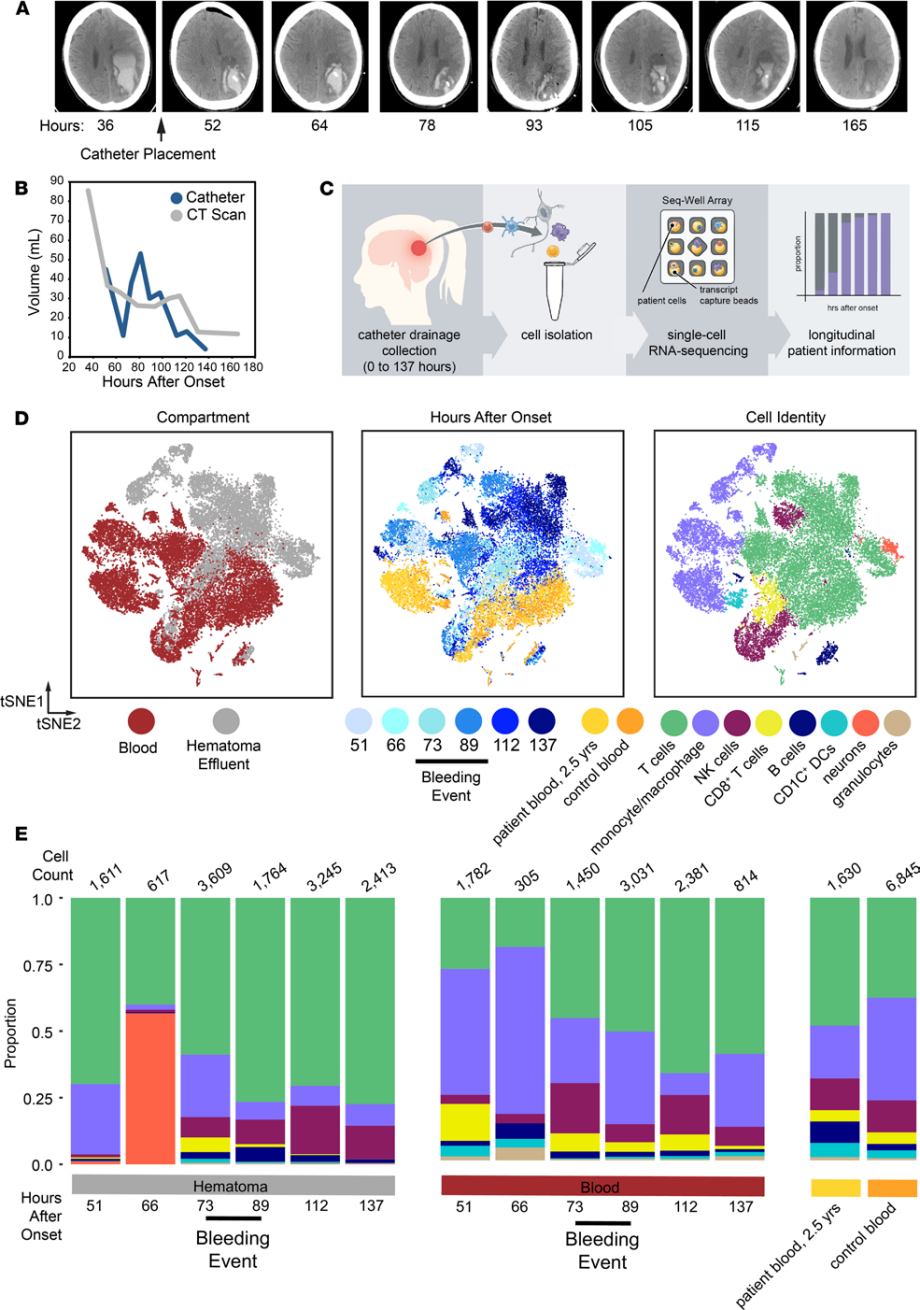
Single-cell RNA-Seq on cells isolated from hematoma effluent and peripheral blood in a living patient over the course of intracerebral hemorrhage. (**A**) Selected CT scans as a function of time after onset (h). The time of catheter placement is indicated. (**B**) Plot of hematoma volume and drainage as a function of time as measured by both CT scan and the volume of hematoma effluent from the catheter in the 8 hours prior to collection. (**C**) Schematic overview of sample collection and processing for generating single-cell RNA-Seq (scRNA-Seq) data from patient hematoma effluent and blood. Single cells from hematoma effluent and blood were isolated, and scRNA-Seq profiles were generated using the Seq-Well platform. (**D**) *t*-Distributed stochastic neighbor embedding (t-SNE) plot along components 1 and 2 for all high-quality single cells (*n =* 24,877 single cells across 7 patient time points, and *n =* 6845 single cells across 4 control donors). The t-SNE plot is colored by compartment of origin (left), time after onset (middle), or cell identity (right). Cell identity was determined by shared nearest-neighboring clustering, marker selection, and module scoring (Supplemental Figure 2). The estimated onset of the asymptomatic rebleeding event is indicated with a black bar and is based on changes detected in CT scan, catheter drainage data, and sequencing data. (**E**) Stacked frequency plots for each indicated condition and colored by identified cell type. Estimated earliest time point of rebleeding event is indicated. Total cell counts per cluster are reported directly in Supplemental Table 3.

A 74-year-old female with hypertension presented to the emergency room with difficulty speaking and right-sided weakness (Supplemental Table 1; supplemental material available online with this article; https://doi.org/10.1172/jci.insight.145857DS1). Her initial NIH Stroke Scale Score (NIHSS) was a 19, and symptoms included aphasia and right-sided hemiplegia, sensory loss, and visual field deficit. A CT scan of the brain showed a large left ICH. Two days after ICH onset, she was enrolled into the MISTIE III trial and randomized to the surgical arm. At 51 hours after hemorrhage, 45 mL liquid hematoma was aspirated and the drainage catheter was placed. The following day, instillation of tPA (1 mg every 8 hours) was initiated per trial protocol with subsequent effective drainage of the remaining hematoma (Figure 1A). Beginning on day 3 after hemorrhage, an increasing volume of drainage was noted from the catheter and tPA was discontinued. However, the patient consistently improved neurologically, and the catheter continued to drain the hematoma for 2 more days before being removed. The patient was subsequently discharged to an acute rehabilitation facility. Thirty days after ICH onset, she had improved to mild aphasia (difficulty speaking), right visual field deficit, and mild right hemiparesis (weakness) and continued to recover functionally. At 2.5 years after onset, she was active with minimal aphasia and right-sided vision loss (NIHSS 3).

During the patient’s hospital stay, her hematoma was effectively drained (Figure 1A) and she improved clinically. Midway through her treatment, increasing hematoma volume by CT scan and an increase in hematoma drainage suggested that she experienced an asymptomatic rebleeding event, and treatment with tPA was halted (Figure 1A and Supplemental Table 1). Volumetric quantification by serial CT scans suggests that this event occurred between 93 and 105 hours after hemorrhage onset. However, the volume and cellularity of hematoma drainage began to rise by 73 hours after onset, prior to hematoma expansion, as measured by imaging (Figure 1B and Supplemental Table 1). These data suggest that the patient’s rebleeding event occurred prior to the increased volume seen on CT scan.

### scRNA-Seq characterization of cells from blood and hematoma effluent.

We hypothesized that detailed analysis of the cellular composition of the hematoma effluent in this patient would provide insight into the molecular features of the hematoma microenvironment, as well as her rebleeding event, and determine whether immune responses are brain tissue specific. Using Seq-Well, we performed scRNA-Seq on individual hematoma cells and peripheral blood collected longitudinally during her hospitalization as well as peripheral blood collected after 2.5 years, and control blood from age-matched healthy donors (Figure 1C; ref. 6). We generated single-cell transcriptional profiles for 31,722 cells spanning 7 time points after onset, and these are displayed along with control samples using a *t*-distributed stochastic neighbor embedding algorithm (t-SNE) (Figure 1D and Supplemental Figures 1 and 2) along the first 2 components. Replicates (performed for most time points) clustered together across each time point and have comparable quality control metrics (Supplemental Figure 1). Overall, we found that blood and hematoma-derived cells were transcriptionally distinct at almost all time points, and separated in t-SNE space by compartment, suggesting that the local milieu within the hematoma is a major driver of leukocyte transcriptional state and function after ICH.

After removing red blood cells based on expression of hemoglobin genes, we identified 7 major cell types, including neurons, B cells, T cells, monocytes, DCs, NK cells, and neutrophils (Figure 1D and Supplemental Figure 3, A–C). We found that monocytes/macrophages and T cells were the most abundant in both peripheral blood and hematoma samples. Moreover, the other major immune cell types were consistently identified in every sample, were comparable in quality, and were present in consistent proportions across replicate samples (Supplemental Figure 3, B and D). We confirmed these findings using separately collected longitudinal samples collected from this patient and analyzed by mass cytometry (Supplemental Figure 4 and Supplemental Table 2). Our mass cytometry results further corroborate our scRNA-Seq frequencies, showing that T cells and monocytes were the predominant cell types profiled in both blood and hematoma. Finally, in our scRNA-Seq data, we found a large cluster of neurons that appeared exclusively in hematoma effluent at high frequency prior to increased hematoma drainage at 66 hours (Figure 1, D and E, and Supplemental Table 2). These neurons were the sole cluster positive for KIF5A, a neuron-specific kinesin subunit (Supplemental Figure 3C; refs. 7, 8). At the 66-hour time point, in both blood and hematoma, we obtained fewer cells, which may contribute to some of the variability at this time point. However, we also performed bulk RNA-Seq analysis on sorted populations of immune cells, including CD4^+^ T cells and monocytes (Supplemental Figure 5, A and B), from this patient’s blood and hematoma at several time points (Supplemental Table 1). These data, combined with a hallmark gene set enrichment analysis (Supplemental Figure 5C), corroborates substantial transcriptomic variation over time despite little variability in broad immune cell frequencies assessed by flow cytometry (data not shown).

### Comparison to control donor blood and patient follow-up blood.

To assess whether the leukocyte populations identified in the peripheral blood after ICH differed from baseline, we also generated single-cell transcriptional profiles from the patient’s blood collected 2.5 years after ICH and from the blood of 4 age-matched healthy donors (Figure 1, D and E). The follow-up blood and healthy control blood generally overlap and may suggest an overall return to baseline in circulating leukocytes in the blood of this patient (Supplemental Figure 2).

### Myeloid cell subclustering analysis.

In the MISTIE III trial, 2% of patients in the surgical arm had a symptomatic rebleeding event; however, 32% had asymptomatic rebleeding (5, 9). Hematoma effluent volume drainage is an imperfect measurement of rebleeding, as some hematoma cavities have variable connections with the ventricular or subarachnoid space and, at times, volume may increase owing to CSF drainage. This event is difficult to identify or predict at the bedside. The implications of rebleeding on the local immune response and long-term outcomes remain unknown but have an important impact of treatment (i.e., cessation of tPA). We hypothesized that a deeper analysis of the predominant cell types in our scRNA-Seq data, including T cells and myeloid cells, could better delineate the compartment-specific response to an asymptomatic rebleed. To accomplish this, we performed subclustering analyses on each population separately.

**Figure 2:**
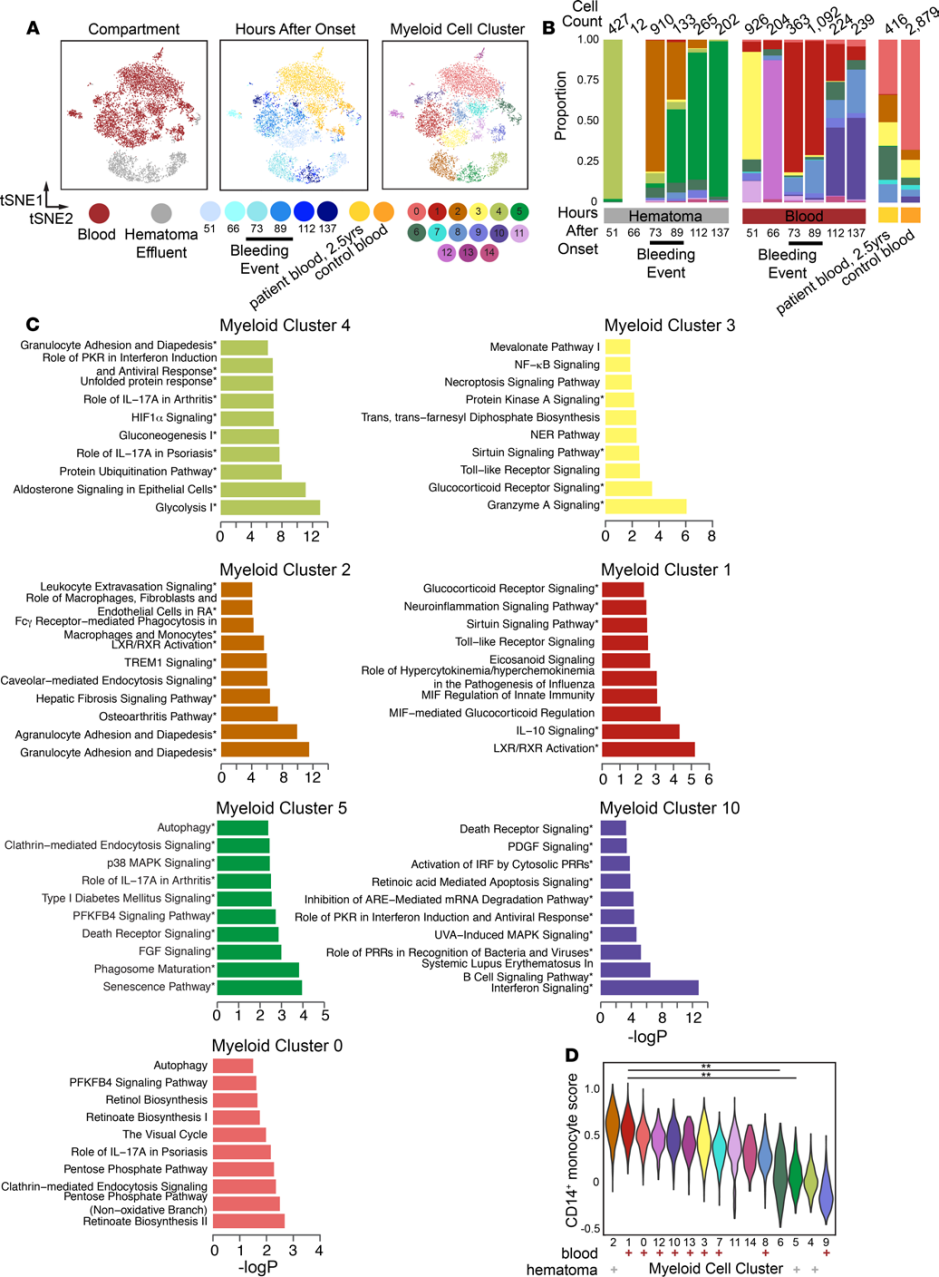
Shifts in prevalence and phenotypes of myeloid cells in hematoma effluent and blood over time. (**A**) t-SNE plot of reclustered monocytes, macrophages, and DCs from Figure 1 (*n =* 8292 cells). The reclustered t-SNE plot is colored by hematoma or blood (left), time after onset (middle), or subcluster identity. (**B**) Stacked frequency plot of new clusters by hours after onset in hematoma and blood. (**C**) Top 10 significantly enriched IPA pathways for selected clusters. Myeloid subclusters 4, 2, 5 emerge sequentially in hematoma; myeloid subclusters 3, 1, and 10 emerge sequentially in blood; and subcluster 0 predominates in patient follow-up at 2.5 years and control blood. Remaining clusters are presented in Supplemental Figure 8. Pathways with 3 or more molecules in the query gene list are indicated by an asterisk. RA, rheumatoid arthritis; PRRs, pattern recognition receptors. (**D**) Violin plot of module scores for an inflammatory monocyte gene signature for each subcluster. All gene modules scored are presented in Supplemental Figure 9. Each subcluster is annotated as predominantly blood (red +) or hematoma (gray +) in origin below the plot. Annotated adjusted *P* values were calculated using Wilcoxon’s rank-sum test with Benjamini-Hochberg *P* value correction, and only select comparisons are annotated on the plot (**P_adj_ < 0.001). Full pairwise results for each cluster are shown in Supplemental Table 7.

Among myeloid cells (macrophage, monocyte/macrophage, and DCs; ref. 10), we found that separation by compartment (i.e., blood vs. hematoma) was preserved (Figure 2A) and identified 14 subclusters, as well as defining genes for each (Figure 2A, Supplemental Figure 6A, and Supplemental Table 4). Three of these (myeloid subclusters 2, 4, and 5) were almost exclusively found in hematoma effluent (Figure 2B and Supplemental Figure 6B). We found that the frequency of myeloid subclusters shifted over time, with several appearing in either blood or hematoma around the time of rebleeding (Figure 2B).

Functional gene enrichment analyses on myeloid subcluster defining genes (Supplemental Figure 6C) revealed distinct pathways that were active in each (Figure 2C, remaining clusters in Supplemental Figure 7 and Supplemental Table 5). Blood prior to rebleeding was predominantly composed of myeloid subcluster 3 at 51 hours (enriched for granzyme signaling and glucocorticoid signaling pathways) and myeloid subcluster 12 at 66 hours (enriched for iron homeostasis and several T cell interaction pathways). Blood during the rebleed as detected by catheter drainage (73–89 hours) was predominantly composed of myeloid subcluster 1, enriched for liver X receptor/retinoid X receptor activation and IL-10 signaling, and then by myeloid subcluster 10, enriched for IFN signaling, at 112 and 137 hours. Subclusters unique to control and the patient’s long-term follow-up blood (myeloid subclusters 0 and 13, Supplemental Figure 6B) were enriched for autophagy. Overall, subclusters predominantly in blood were enriched for a spectrum of pathways involved in innate immune cell functions that changed over time, including TLR signaling, glucocorticoid signaling, EIF2 signaling, and IFN signaling.

Hematoma prior to rebleeding was predominantly composed of myeloid subcluster 4, which was enriched for glycolysis and HIF-1α signaling. We only recovered 12 myeloid cells at 66 hours in hematoma, limiting our resolution at this time point. Then, a time-dependent shift occurred from dominance by myeloid subcluster 2 to myeloid subcluster 5. Myeloid subcluster 2 was enriched for pathways related to myeloid cell extravasation and tissue infiltration, possibly indicating new infiltration after rebleeding (Figure 2C), and myeloid subcluster 5 was enriched for pathways related to tissue repair and senescence, including phagosome maturation, FGF signaling, autophagy, and senescence. This shift suggests transitions between dominant clusters showing innate activation and recruitment to cell states involving phagocytosis and perhaps functions related to the resolution of inflammation. Taken together, we found alterations in the overall transcriptional states of myeloid cells in both blood and hematoma, and gene set enrichments suggest these cells, particularly in hematoma, may have differing functions altered by their unique physiological niche that shift over time.

### Myeloid subcluster module scoring analysis.

To better contextualize the phenotypes of myeloid subclusters, we performed module scoring against a list of curated human monocyte/macrophage gene sets (Supplemental Table 6, Supplemental Figure 8A. refs. 11–14). We performed clustering and principal component analysis (PCA) on these scores to identify modules that explain differences across myeloid clusters. We found that most of these known signatures did not explain differences between myeloid subclusters (Supplemental Figure 8A). Nonetheless, we identified 4 gene signatures that predominantly explain differences between myeloid subclusters that were either hematoma or blood in origin; these signatures accurately described blood monocytes/macrophages but not those from hematoma (Supplemental Figure 8B). Specifically, we found that the predominantly blood subclusters (myeloid subclusters 0, 1, 3, 6, and 7–13) and the subcluster that emerged in the hematoma at 73 hours (myeloid subcluster 2) scored similarly for a CD14^+^ monocyte gene signature identified recently in blood (Figure 2D; ref. 12). We additionally scored each myeloid subcluster for gene sets that define border-associated macrophages (BAMs) (15, 16) and microglia (Supplemental Table 6; refs. 17–19). We found that each subcluster, regardless of compartment, scored low for microglia but high for BAM and CD14^+^ signatures (Supplemental Figure 9). This suggests that, aside from the *KIF5A*^+^ neuronal cell cluster, most cells in hematoma effluent are peripheral in origin. However, future work will be necessary to better delineate the peripheral versus local axis of response to ICH, especially since peripherally derived macrophages adjust their transcriptomes to reflect tissue residence (20). Taken together, our data suggest that myeloid subcluster 2 may represent monocytes newly entered into the hematoma owing to a recent rebleed, whereas other hematoma clusters, such as myeloid clusters 4 and 5, are defined by activation states induced by local signals in the hematoma and do not resemble clusters previously defined in human blood or by in vitro stimulations (12–14). When considered in the context of the timing of the increased draining volume from the catheter and the emergence of a neuron-like cluster at 66 hours, our data may suggest a rebleeding event prior to the clinically identified changes in hematoma volume detected by a daily CT scan.

### T cell subclustering analysis.

**Figure 3:**
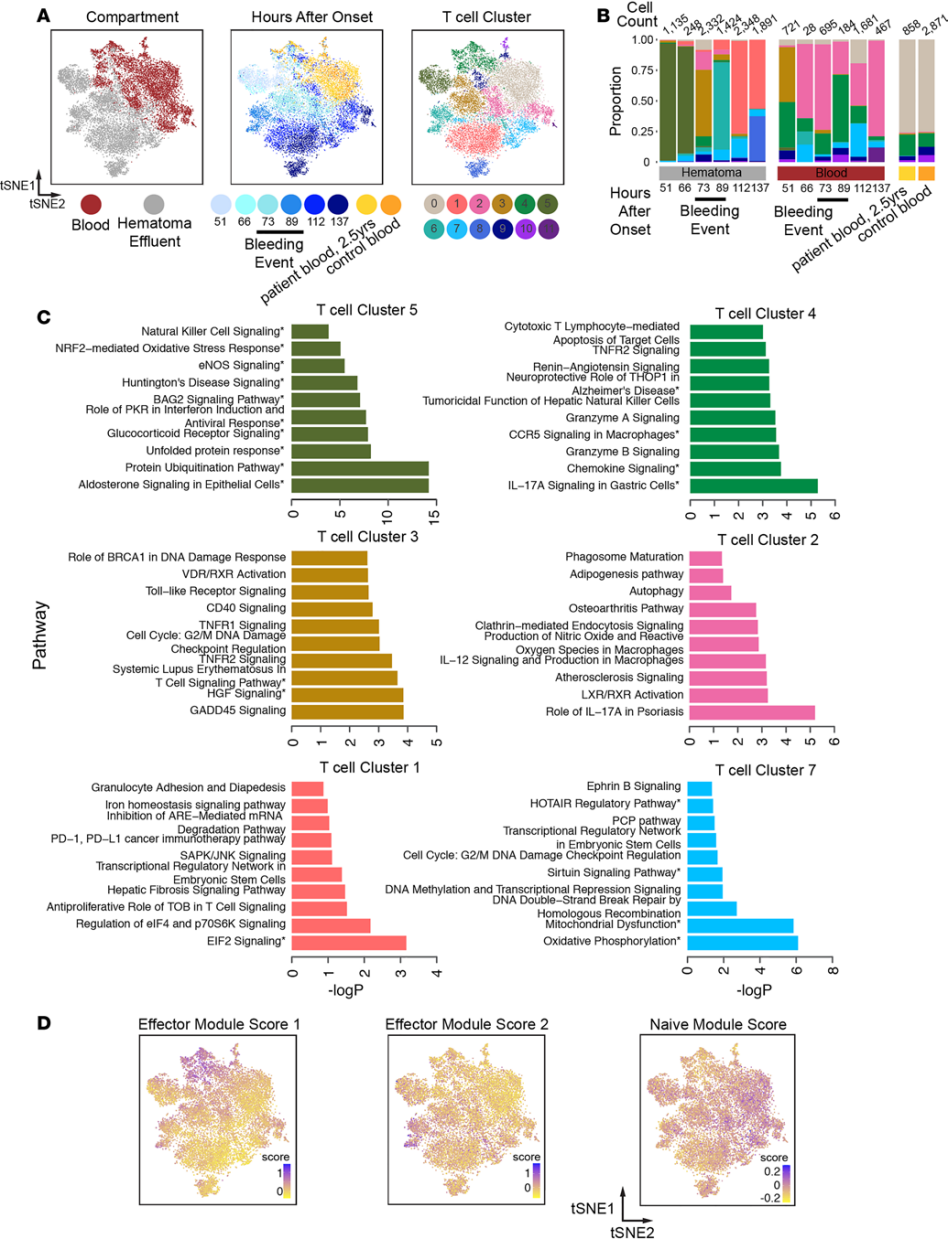
Shifts in prevalence and phenotypes of T cells in hematoma effluent and blood over time. (**A**) t-SNE plot showing reclustered T cells (*n =* 16,883 cells) from Figure 1. The reclustered t-SNE plot is colored by hematoma or blood (left), time after onset (middle), or T cell subcluster identity (right) determined by reclustering analysis. (**B**) Stacked frequency plot of new T cell clusters by hours after onset in blood and hematoma. (**C**) Top 10 significantly enriched IPA pathways for selected clusters. T cell subclusters 5, 3, and 1 emerge in hematoma, and T cell subclusters 4, 2, and 7 are found in blood. Remaining clusters are presented in Supplemental Figure 11. Subcluster 0 was defined by 2 marker genes (*TXNIP* and *LTB*). Pathways with 3 or more molecules in the query gene list are indicated by an asterisk. (**D**) t-SNE plots colored by each indicated module score.

Our data also revealed substantial involvement of T cells in the hematoma at all time points. Subclustering over all hematoma and peripheral blood T cells from the patient and control blood resulted in 12 T cell subclusters that also separated overall by compartment (Figure 3A and Supplemental Figure 10). As with monocytes, we found that T cell frequencies were dynamic over the course of ICH in hematoma (Supplemental Table 8), and these clusters were enriched for various functional pathways (Supplemental Table 9). In the blood, there was an initial shift in the dominant cluster from T cell subcluster 3 to T cell subcluster 2 prior to the rebleed, which then remained highly frequent for the remaining time points (Figure 3B). Several subclusters were also predominantly blood in origin (subclusters 0, 2, 4, 10, and 11). T cell subclusters 2 and 3, which were enriched for IL-17A and GADD45 signaling, respectively, also emerged in the hematoma at 73 hours. The emergence of these clusters in hematoma at this time paralleled the emergence of a more blood-like monocyte subcluster at the same time point, further supporting evidence this was the beginning of the rebleeding.

Several subclusters of T cells were predominantly found in hematoma (Supplemental Figure 10B; e.g., T cell subclusters 1, 3, 5, 6, and 8). T cell subcluster 5, prevalent in early hematoma samples, was enriched for HSPs, protein ubiquitination, and eNOS signaling (Figure 3D and Supplemental Table 8). T cell subcluster 3, which emerged at 73 hours in hematoma, was enriched in GADD45 signaling and several immune activation pathways (Figure 3C, additional clusters in Supplemental Figure 11). T cell subcluster 1, emerging at later time points in hematoma, shows expression of genes involved in transcriptional regulation, anti-proliferation, and iron homeostasis. Interestingly, we did not find clear patterns of chemokine and receptor expression (21) in specific clusters in either myeloid or T cell subclusters; however, we did find elevated CXCR4 expression consistently across myeloid and T cell subclusters (Supplemental Figure 12). Overall, we found that T cells that originated in blood were enriched for innate immune interactions, IL-17 signaling, chemokine signaling, and sirtuin signaling, whereas those predominantly from hematoma were enriched for stress response pathways, diapedesis, and neuroinflammation. Both the patient’s long-term follow-up blood sample and the healthy control blood samples were predominated by T cell subcluster 0, which was defined by only 2 marker genes (*TXNIP* and *LTB*), precluding pathways analysis for this subcluster.

### T cell subcluster module scoring analysis.

To better contextualize our T cell subclustering results, we again performed module-scoring analysis with known T cell gene signatures previously derived from human transcriptional data (Supplemental Table 10). Unsupervised clustering, followed by PCA on module scores, revealed several modules that describe variation across T cell subclusters (Supplemental Figure 13A). We found that 3 gene modules that describe effector functions in T cells drove major axes of variation in our data set (Figure 3D). One signature is associated predominantly with T cell subcluster 4 and is defined by T cell effector genes like *GNLY*, *CCL4*, and *GZMK* (effector module score 1, Supplemental Figure 13B). The other is associated with T cell subcluster 6, which is a hematoma cluster emerging during rebleeding, and is defined by T cell effector genes like *CXCR4*, *TXN*, and *CSTB* (effector module score 2, Figure 3D, and Supplemental Figure 13B). Generally, cells in clusters on the right in the t-SNE plot shown in Figure 3D, including T cell subclusters 0, 2, and 7 (found predominantly in blood), score higher for a naive T cell module. Overall, these data, combined with our pathway enrichment results, suggest that T cells in the predominantly hematoma-derived clusters may be enriched for unique T cell functions, including several related to effector functions and stress responses.

## Discussion

Based on the emergence of several blood-like monocyte and T cell subclusters in the hematoma at 73 hours, as well as increased drainage volume and cellularity, we estimate that the time of rebleeding was between 66 and 73 hours after ICH onset, a full day prior to detection by CT scan. It is also intriguing that neurons emerged in the hematoma effluent immediately prior to this event, but the consequences of this is unknown as the patient continued to improve. Monitoring of specific peripheral blood biomarkers found in effluent could thus potentially enable earlier detection of a rebleeding event to guide a more effective treatment paradigm. Additionally, the emergence of myeloid subcluster 12 in the blood at the 66-hour time point would suggest that peripheral monitoring in a larger cohort may shed light on peripheral changes in response to rebleeding. Although our study presents only 1 patient’s ICH trajectory, it suggests that large-scale scRNA-Seq studies could provide more insight into specific blood cell states that may better inform patient treatment and outcome and highlight pathways for future research.

Our transcriptional analyses of leukocytes over the course of ICH in this patient suggest coordinated, dynamic stages of monocyte and T cell responses within the hematoma that were altered after asymptomatic rebleed, indicating rapid adaptation and response to local changes in the hematoma tissue milieu. Interestingly, we did not see an exact return to the cell states that characterized the 51-hour time point. This suggests that the response may be different to a secondary bleed, or we simply were not able to sample the hematoma long enough to determine if the cells at 137 hours were on a trajectory back to what they looked like prior to rebleeding. Additionally, we studied the cellular composition of the blood of this patient more than 2 years after ICH, and our data suggest that the peripheral blood of this patient, who had an excellent outcome, is comparable to that of age-matched controls.

The dynamics of leukocyte infiltration and activation after ICH have been difficult to study in patients, especially at the site of injury (22). At present, our understanding is derived primarily from rodent models and peripheral measures; how the kinetics in these models compare to those in humans is unknown (23, 24). Using the infrastructure of the MISTIE III trial, for the first time to our knowledge we characterized the local cellular response to ICH and an associated rebleeding event in the brain of a living patient at single-cell resolution. We characterized thousands of single cells from both blood and hematoma effluent at several points after ICH onset, allowing us to identify cellular phenotypes that were dynamic in both the T cell and myeloid lineages. Notably, the emergence of myeloid subcluster 2 and T cell subcluster 2 in the hematoma after rebleeding, followed by the sequential emergence of new cell states, suggests that newly infiltrating immune cells adapt transcriptionally to dynamic injury events in the brain.

tPA was administered to assist the hematoma drainage in the trial. In a separate analysis, we found no significant gene expression changes in monocytes or granulocytes after ex vivo incubation with tPA (25). This suggests that tPA alone likely does not impact directly the transcriptome of innate immune cells. However, further work will be necessary to determine the in vivo impact of tPA on further analyses of immune cells, and more specifically, how alterations in the clot as a result of tPA treatment might impact these cells.

Our work plays a crucial role in demonstrating how rapidly immune cell activation states change after brain injury in humans and sets the stage for framing critical time periods for future studies. Although this case study is small, our data provide proof of principle that biological investigations in collaboration with clinical trials can provide insight into potential therapeutic pathways to target to minimize brain injury. This raises the exciting possibility that, in the near future as personalized medicine evolves, this and other approaches could be used clinically to interrogate cellular responses and uncover meaningful clinical events in patients to help guide treatment decisions. However, more work is needed to further explore this potential.

## Methods

### Patient selection.

The ICHseq trial substudy collected samples for RNA-Seq on sorted leukocyte populations. After the first surgical patient was enrolled at Yale, we developed a protocol to collect an additional sample daily for scRNA-Seq, leading to the analysis of this patient. The trial was completed before an additional patient was randomized to the surgical arm of the trial at Yale, prohibiting additional patients in this analysis. Control donors (*n =* 4, 2 men and 2 women, ages 63–93) were recruited, and samples were also collected under an IRB-approved protocol.

### Patient hematoma effluent and blood collection and processing.

Each sample was centrifuged at 500*g* for 10 minutes, and the supernatant was removed. Then, each sample was resuspended in HBSS (Gibco) and treated with 2.5 units/mL Benzonase (MilliporeSigma, E1014) for 10 minutes at room temperature. Samples were then passed through a 70 μm filter to remove tissue debris, washed with 25 mL HBSS, and centrifuged at 300*g* for 10 minutes. After supernatant was removed, platelets and most erythrocytes were removed from these samples using a LeukoLOCK filter (Life Technologies) as previously described. Briefly, after the sample passed through the filter binding leukocytes, the filter was backflushed to recapture the leukocytes. This backflush was centrifuged at 300*g* for 8 minutes, and supernatants were removed until the mixed leukocyte/erythrocyte cell pellet composed approximately 50% of the total volume of the sample. Granulocytes were removed from this sample using the RosetteSep Human Granulocyte Depletion Cocktail (STEMCELL Technologies) and subsequent Ficoll density gradient centrifugation according to the manufacturer’s protocol. Finally, the resulting cell pellets, which still contained trace numbers of erythrocytes, were subjected to 2 rounds of erythrocyte lysis at room temperature for 5 minutes each using BD Pharm Lyse buffer, washed, and resuspended in RPMI medium containing 10% FBS.

### Generation of scRNA-Seq data with Seq-Well.

Seq-Well was performed as described previously (6). About 15,000 cells were loaded onto each array preloaded with uniquely barcoded mRNA capture beads (ChemGenes). Arrays were washed with protein-free RPMI media, then sealed with polycarbonate membranes. Arrays were incubated at 37°C for 30 minutes to allow membranes to seal, then transferred through a series of buffer exchanges to allow for cell lysis, transcript hybridization, bead washing, and bead recovery from arrays after membrane removal. Reverse transcription was performed with Maxima H Minus Reverse Transcriptase (Thermo Fisher Scientific), excess primers were removed using an Exonuclease I digestion (New England Biolabs), and whole transcriptome amplification (WTA) by PCR was performed using KAPA Hifi PCR Mastermix (Kapa Biosystems). WTA product was purified using Agencourt AMPure beads (Beckman Coulter) and 3′ digital gene expression (DGE) sequencing libraries were prepared using Nextera XT (Illumina). Two sequencing libraries’ arrays were sequenced per NextSeq 500/550 run using a 75-cycle v2 sequencing kit (Illumina) with a paired-end read structure (R1: 20 bases; I: 8 bases; and R2: 50 bases) and custom sequencing primers.

### scRNA-Seq computational analyses.

Raw sequencing data were demultiplexed and aligned to the Hg19 genome using publicly available scripts on Terra (scCloud/dropseq_workflow, version 11, https://app.terra.bio/). The resulting UMI-collapsed DGE matrices were used as input to Seurat (v3) for further analyses in R (v3.6.2). Initial clustering was performed, and on the basis of marker genes identified using Seurat’s Wilcoxon rank-sum test, red blood cells were identified by hemoglobin gene expression and excluded from further analysis, resulting in a total of 31,722 cells across all conditions. All data were normalized, the top 2000 variable genes were identified, and the data were clustered at a resolution of 2. Marker genes for each cluster were identified using Seurat’s Wilcoxon rank-sum test, broad immune cell types were labeled by comparing with known marker genes that have been previously published, and clusters were collapsed into cell identity labels (Figure 1; refs. 12, 13).

For subclustering analyses, T cells or myeloid cells were analyzed separately. Each cell type was renormalized, the top 2000 variable genes were identified, and the data were clustered across several resolutions to identify resolutions that produced nonredundant clusters (resolution = 0.6 for T cell subclustering, and resolution = 0.6 for myeloid subclustering) as determined by marker gene identification using Seurat’s Wilcoxon rank-sum test. Notebooks to reproduce all analyses performed in R are available for download (commit ID 7698288513c7c666dbee1d708c8cb07ca51308dc; https://github.com/bagoods/ICH-seq_scRNAseq_Analysis).

Functional enrichment was performed using Ingenuity Pathway Analysis (Qiagen, version 47547484) using adjusted *P* values of significant genes in each cluster as input, and significant canonical pathways were reported if their *P* value was significant (*P*_adj_ > 0.05). If 3 or more molecules of the pathways were identified in the input gene lists, these pathways are bolded in the main manuscript figures. For creating gene module scores, gene lists were manually curated from existing literature sources (Supplemental Tables 6 and 10), and scores were generated in R using Seurat.

### Mass cytometry sample preparation.

Cell suspensions from each time point were stained for mass cytometry and fixed immediately upon isolation (26). Antibody mixes (Supplemental Table 2) were prepared fresh each day in BSA stain buffer (BD Biosciences, 554657) and centrifuged through a 0.1 μm filter (MilliporeSigma, UFC30VV00) at 12,000*g* for 4 minutes; this process reduced nonspecific labeling of cells with free isotopes. Up to 3 × 10^6^ cells in RPMI containing 10% FBS were transferred to a 1.5 mL Eppendorf tube and washed with PBS. Cell suspensions were centrifuged at 300*g* for 8 minutes, and supernatant was removed from the cell pellet. For all centrifugation steps, a fixed rotor microcentrifuge was used, and supernatants were carefully removed by pipette. To mark cells for viability, cell pellets were resuspended in 1 μM Cell-ID cisplatin (Fluidigm, 201194) and incubated for 3 minutes at room temperature. Cells were washed with 1 mL BSA stain buffer and centrifuged at 300*g* for 8 minutes, and supernatant was carefully removed. Cell pellets were resuspended in antibody mix and incubated on ice for 40 minutes. Cell pellets were washed with 1 mL BSA stain buffer, centrifuged, resuspended in Fix/Perm buffer from the FoxP3 staining kit (eBioscience, 00-5523-00), and incubated overnight at 4°C. The next morning, samples were centrifuged at 800*g* for 5 minutes, resuspended in 300 μL BSA stain buffer, and stored at 4°C until all time points had been collected. Then, all samples were centrifuged and resuspended in 125 nM Cell-ID Intercalator-IR (Fluidigm, 201192A) diluted in Fix/Perm buffer, and incubated overnight at 4°C. Immediately before running each sample on the mass cytometer, that sample was centrifuged, supernatant was removed, and the pellet was resuspended in deionized water.

### Data analysis for mass cytometry.

Initial gating was performed to identify CD45^hi^ cisplatin^lo^ viable leukocytes, after first using iridium intercalator to exclude doublets and noncellular debris. Cells meeting these criteria were then normalized using bead standards to account for variations in cytometer sensitivity as previously described (26). Cell populations were identified using FlowJo software (v10, Tree Star Inc.) according to the following marker combinations for each: CD4^+^ T cells (CD3^+^CD19^–^CD4^+^), CD8^+^ T cells (CD3^+^CD19^–^CD8α^+^), NK cells (CD3^–^CD56^+^CD11b^lo^), B cells (CD3^–^CD56^–^CD11b^–^CD19^+^), monocytes (CD3^–^CD56^–^CD11b^hi^CD19^–^CD66a^–^CD14^+^CD11c^+^), and neutrophils (CD3^–^CD56^–^CD11b^hi^CD19^–^CD66a^+^HLA-DR^lo^).

### Bulk RNA-Seq sample preparation and analysis.

Up to 5 × 10^6^ leukocytes isolated from each sample as described in *Patient hematoma effluent and blood collection and processing* were used for sorting. Cells were incubated in 1 mL Cell Cover (Anacyte, 800-250) on ice for 10 minutes. CD3^+^ cells were isolated from the PBMC preparation by magnetic selection (STEMCELL Technologies, 17851) according to the manufacturer’s protocol. CD3^+^ cells were stained for flow cytometry using viability dye (Life Technologies, L34972) and the following antibodies: CD45 (Tonbo, 35-0459-T100), CD25 (BD Biosciences, 555432), CD8 (Tonbo, 60-0088-T100), CD127 (BioLegend, 351316), CD2 (BD Biosciences, 562638), CD4 (Tonbo, 75-0049-T100), CD11b (Tonbo, 30-0112-U500), CD20 (BioLegend, 302349), and CD56 (BioLegend, 318320). CD45^hi^ CD11b^–^ CD56^–^ CD20^–^ CD2^+^ CD4^+^ CD8^–^, CD127^hi^ CD25^–^ live cells were sorted for CD4 T cell library construction. CD3^–^ cells were stained for flow cytometry with viability dye and the following antibodies: CD45 (Tonbo Biosciences, 50-0459-T100), CD11b (Tonbo, 35-0118-T100), CD14 (BioLegend, 301820), CD16 (BD Biosciences, 560474), CD66a/c/e (BioLegend, 342310), CD2, (BioLegend, 300204), CD20 (BioLegend, 302349), and CD56 (BioLegend, 318320). CD45^hi^ CD2^–^ CD56^–^ CD20^–^ CD66^–^ CD11b^hi^ CD14^hi^ CD16^lo^ cells were sorted for monocyte library construction. Monocyte and CD4 populations were sorted (Supplemental Figure 5, A and B) using a FACSAria II into RNA lysis buffer (Macherey-Nagel, RA1).

RNA-Seq libraries were generated as previously described (27). Briefly, RNA was extracted using the NucleoSpin RNA XS Kit (Macherey-Nagel, 740902) according to the manufacturer’s instructions. Smart-Seq2 cDNA synthesis (28) was performed with the following modifications: a) input RNA was normalized by diluting to approximately 1000 cells per reaction, and b) reverse transcription was performed with Superscript III (Thermo Fisher Scientific, 18080-085) according to the manufacturer’s instructions. Paired-end sequencing libraries were prepared using the Nextera XT (Illumina, FC-131). Libraries were pooled and sequenced on a NextSeq 500/550 (Illumina) using a 75-cycle v2 sequencing kit. Following sequencing, BCL files were converted to merged, demultiplexed FASTQs. These were then mapped to the UCSC hg19 genome using STAR and RSEM. SSGSEA was performed with the Hallmark gene sets using the “SSGSEAProjection” module version 9.1.1 on the GenePattern website (https://www.genepattern.org/, parameters: weighting exponent = 0.75, minimum gene set size = 10, and combine mode = combine.add). Heatmaps of SSGSEA results were created with Morpheus.

### Data availability.

Raw sequencing and processed data for this study have been made available through Gene Expression Omnibus concurrent with publication. Bulk RNA-Seq data for sorted monocytes is available at GSE163256, and all scRNA-Seq and bulk CD4^+^ T cell RNA-Seq are available at GSE166638.

### Statistics.

Statistical analyses for comparing cell frequencies obtained with CyTOF or Seq-Well were performed using GraphPad Prism (version 8.4.3). These data were analyzed using repeated measures 1-way ANOVA followed by post hoc test (Kruskal-Wallis, nonparametric), and *P* < 0.05 was considered statistically significant. For comparison of module scores, analyses were performed in R (version 3.6.2). Module scores between myeloid subclusters were compared by Kruskal-Wallis rank-sum test followed by pairwise comparisons using Wilcoxon’s rank-sum test with Benjamini-Hochberg *P* value correction.

### Study approval.

The patient was enrolled in the MISTIE III trial (NCT01827046) under an IRB-approved protocol including sample collection for the substudy ICHseq. Control donors were recruited, and samples were also collected under an IRB-approved protocol. The Yale IRB is Yale Human Investigations Committee, New Haven, Connecticut, USA. Consent was obtained by subjects or legally authorized representatives prior to participation.

## Author contributions

LHS designed the study in consultation with DFH, JCL, BAG, MHA, and AKS. LHS, AKS, and JCL supervised the study at all stages. Sample processing was performed by MHA and HEB. BAG, MHA, JHD, and NHP performed Seq-Well and associated sequencing with assistance from IF, EM, and RSD. BAG performed transcriptional analysis with input from LHS, MHA, JCL, and AKS. MHA performed mass cytometry and mass cytometry analysis. DFH, IAA, and MZ led the MISTIE III trial internationally and provided trial data for this study, the ICHSeq Investigators provided patient samples for the ancillary study, and CCM and LHS led trial at Yale and provided patient samples and additional data. BAG, MHA, JCL, AKS, and LHS wrote the manuscript with critical input from all authors.

## Supplementary Material

Supplemental data

## Version 1 03/22/2021

Electronic publication
