# Supplementary material for: Leukocyte dynamics after intracerebral hemorrhage in a living patient reveal rapid adaptations to tissue milieu
Source: JCI Insight. 2021 Mar 22;6(6):e145857. doi: 10.1172/jci.insight.145857 (PMC8026179; doi:10.1172/jci.insight.145857)
Supplement: Supplemental data [file jciinsight-6-145857-s115.pdf]

Supplemental Appendix

Table of Contents

Author Lists…………………………………………………….…..2-3

Supplemental Figures ……………………………….……..4-20

Supplemental Tables ………………………………….……21-41

Supplemental References…………………………………..42

Brittany A. Goods, PhD[1,2*], Michael H. Askenase, PhD[3*], Erica Markarian[4], Hannah E. Beatty[3], Riley Drake[1], Ira Fleming[1], Jonathan H. DeLong[3], Naomi H. Philip[5], Charles C. Matouk, MD[6], Issam A. Awad, MD[7], Mario Zuccarello, MD[8], Daniel F. Hanley, MD[9], J. Christopher Love, PhD[2,4,10,#], Alex K. Shalek, PhD[1,2,4,11,12,13,14,#], and Lauren H. Sansing, MD, MS[3,15,#], on behalf of the ICHseq Investigators

[1]Department of Chemistry and Institute for Medical Engineering & Science, Massachusetts Institute of Technology

[2]Broad Institute, Harvard University & Massachusetts Institute of Technology

[3]Department of Neurology, Yale University School of Medicine

[4]Koch Institute for Integrative Cancer Research, Massachusetts Institute of Technology

[5]Department of Immunobiology, Yale University School of Medicine

[6]Department of Neurosurgery, Yale University School of Medicine

[7]Department of Neurosurgery, University of Chicago

[8]Department of Neurosurgery, University of Cincinnati

[9]Brain Injury Outcomes Division, Johns Hopkins School of Medicine

[10]Department of Chemical Engineering, Massachusetts Institute of Technology

[11]Ragon Institute, Harvard University, Massachusetts Institute of Technology, & Massachusetts General Hospital

[12]Division of Health Science & Technology, Harvard Medical School

[13]Program in Computational & Systems Biology, Massachusetts Institute of Technology

[14]Department of Immunology, Massachusetts General Hospital

[15] Human and Translational Immunology Program, Yale School of Medicine

*equal

[#]equal

Corresponding Authors: Lauren Sansing, lauren.sansing@yale.edu, Alex K. Shalek, shalek@mit.edu, J. Christopher Love, clove@mit.edu

ICHSeq Investigators

Site	Principal Investigator	Lead Coordinator
Abington	Larami MacKenzie	Karin Jonczak
Barrow	Peter Nakaji	Norissa Honea
Duke	Michael James	Erlinda Yeh
Hennepin	Thomas Bergman	Kathy France
INOVA	James Leiphart	Tricia Brannan
Johns Hopkins	Wendy Ziai	Vikram Madan
Kansas U Medical Center	Paul Camarata	Jason Gorup
Mayo Jacksonville	Ronald Reimer	Emily Edwards
Miami Valley	John Terry	Angie Shoen
Northwestern	Babak S. Jahromi	Byron Yip
Rush	George Lopez	Becky Holtz
Rutgers	Igor Rybinnik	Michelle Moccio
St Lukes	Darren Lovick	Kelsey Titus
Thomas Jefferson	Jack Jallo	Kara Pigott
U Alabama Birmingham	Mark Harrigan	Lisa Nelson
University of Michigan	Ventatakrishna Rajajee/Aditya Pandey	Ron Ball
U New Mexico	Andrew Carlson	Amal Alchbli
U Louisville	Robert F. James	Ann Jerde
U Utah	Safdar Ansari	Joshua Letsinger
Yale	Kevin Sheth/Lauren Sansing	Linda Ryall

BIOS and MTI:M3 Investigators

Johns Hopkins	Paul Nyquist
Mass General	Josh Goldstein
Johns Hopkins	Vikram Madam
Johns Hopkins	Christina Grabartis
Johns Hopkins	Tracey Economas
Johns Hopkins	Wendy Ziai
Manchester	Adrian R. Parry-Jones

Supplemental Figures

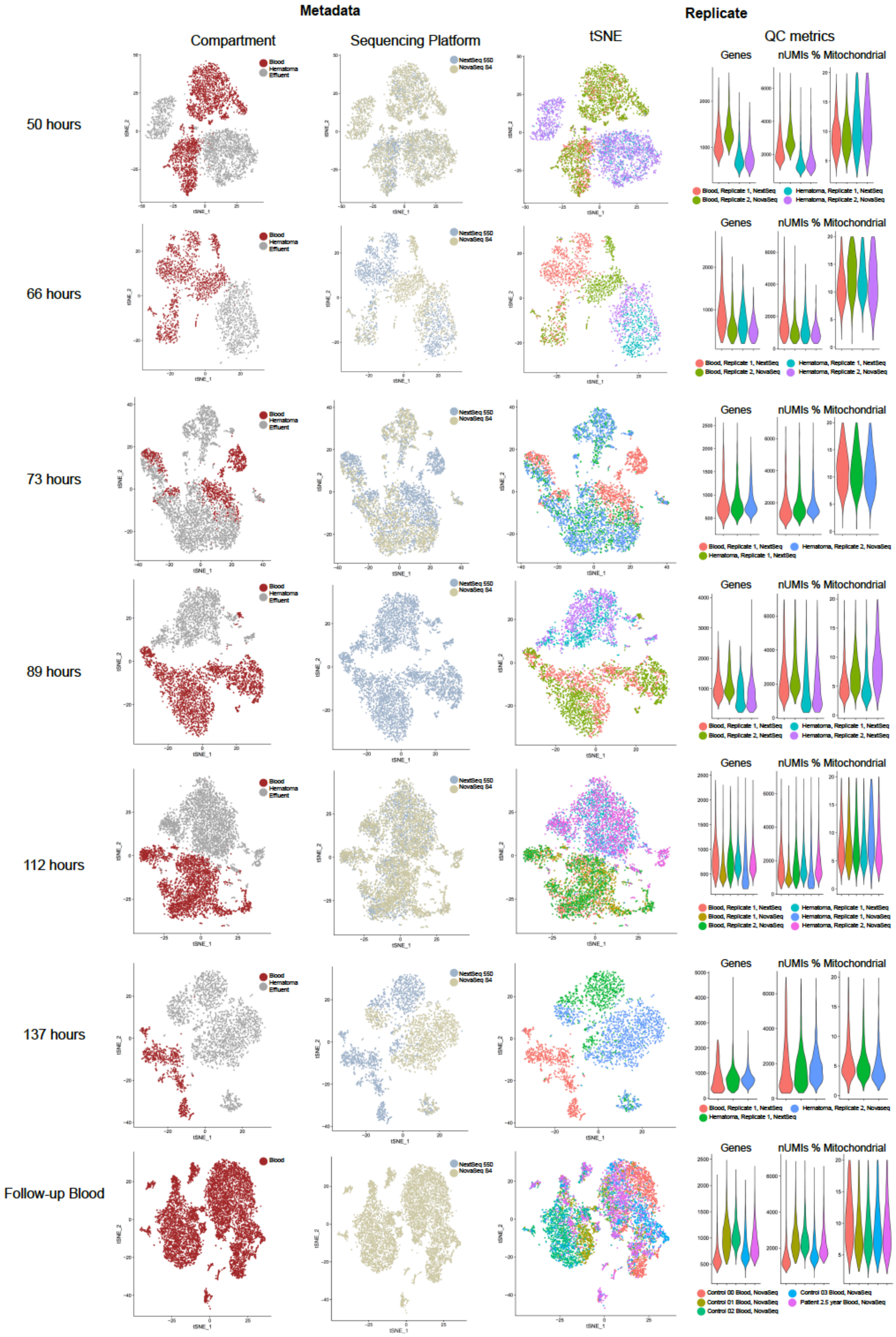

Supplemental Figure 1. Reproducibility across replicates. tSNEs of data for each time point post ICH are shown and colored by metadata (compartment or sequencing

platform) and replicatearray. Violin plots of quality control metrics including number of unique molecular identifiers (nUMIs), number of genes detected (Genes), and fraction of mitochondrial genes for each SeqWell array.

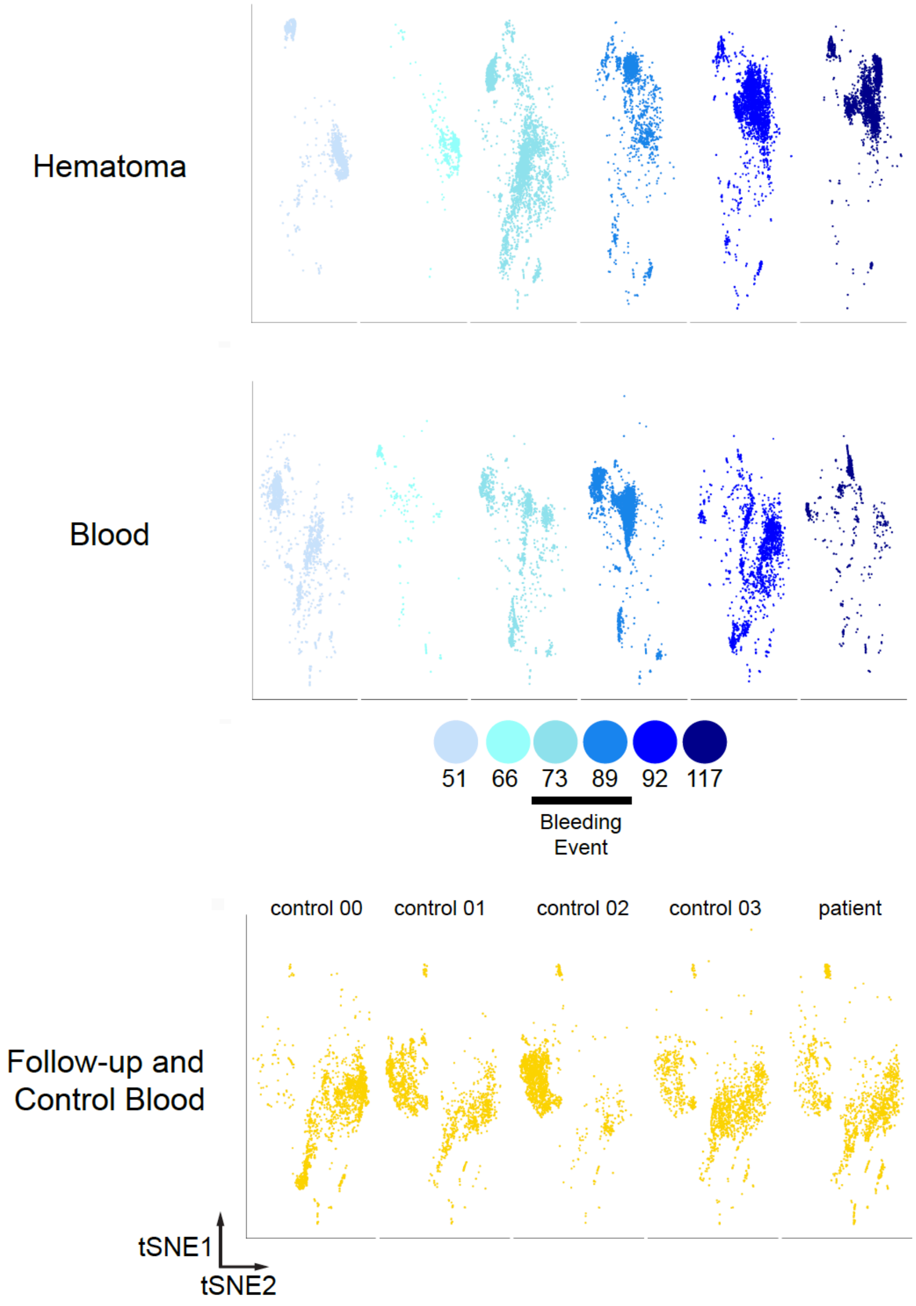

Supplemental Figure 2. tSNE of all data shown by hematoma (top), blood (middle), or control and patient 2.5 year follow-up. tSNEs are also shown by individual time point, showing cell yield for each array.

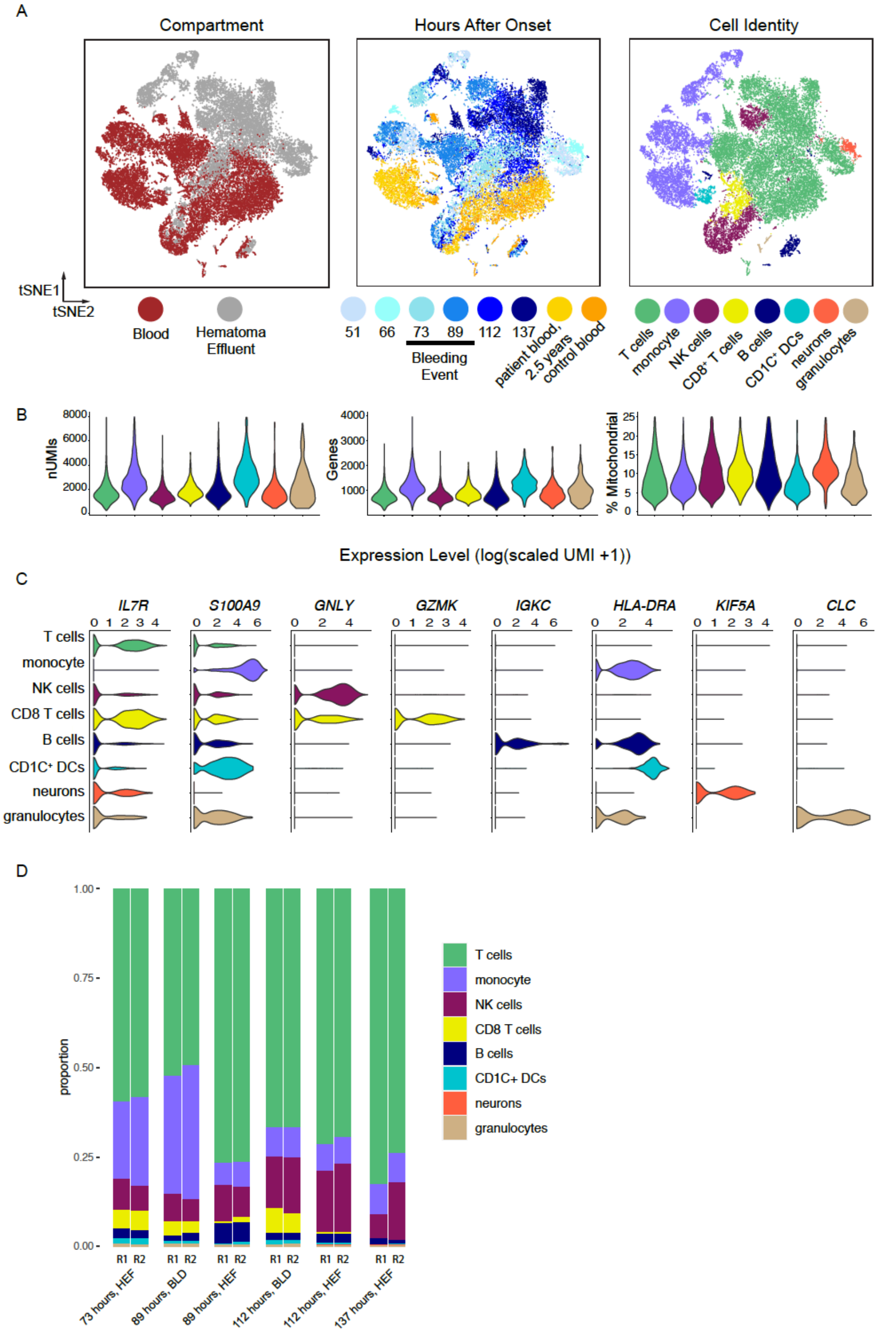

Supplemental Figure 3. tSNE of all data clustered including all cell clusters with key cell type identification genes as feature plots. **A.** tSNE colored by compartment, time, and cell type reproduced from Figure 1. **B.** Violin plots of quality control metrics including number of unique molecular identifiers (nUMIs), number of genes detected

(Genes), and fraction of mitochondrial genes per cell plotted by cell type. **C**. Violin plots show increased expression of selected marker genes for each cluster. **D.** Frequency of each cell type is shown for replicate arrays.

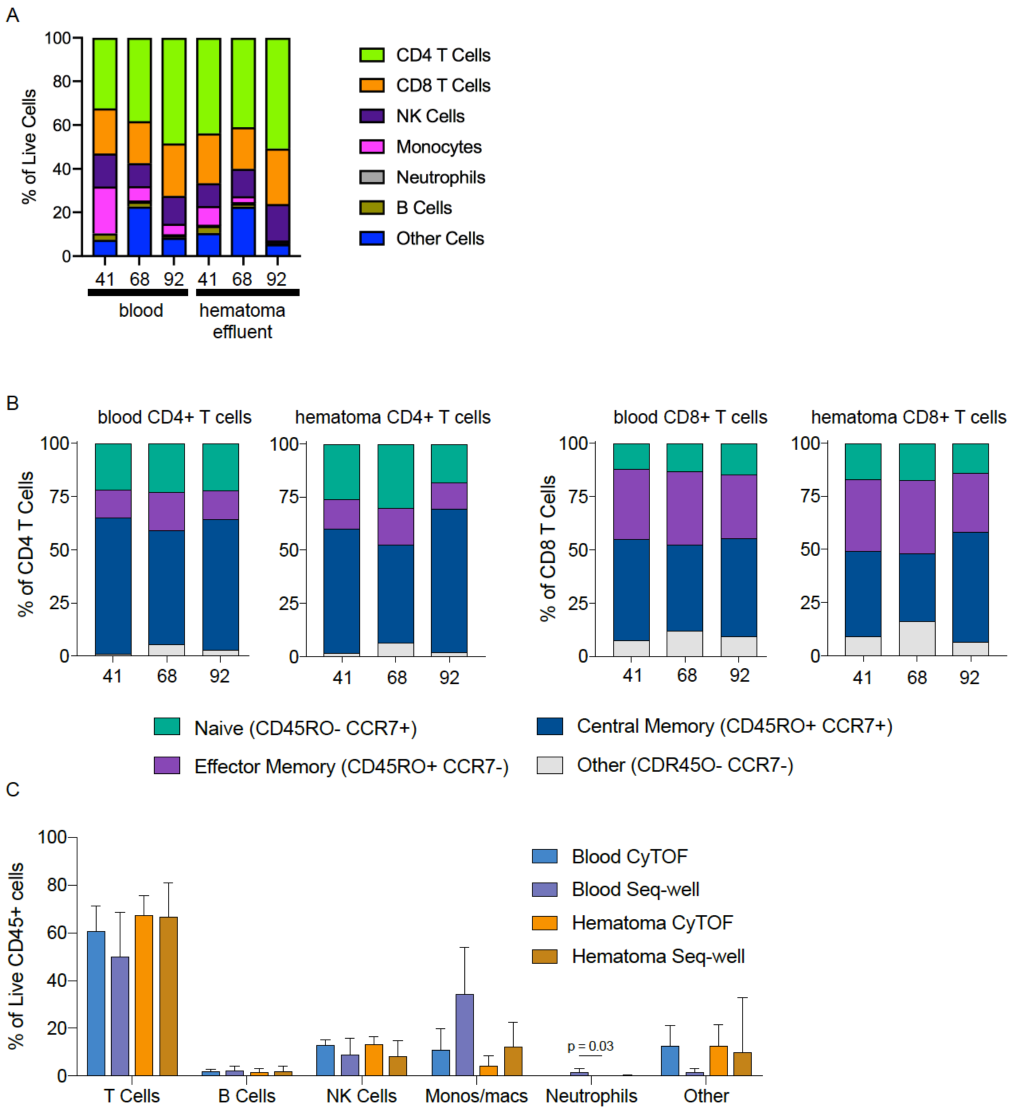

Supplemental Figure 4. Mass cytometry analysis of hematoma and blood. **A**. Stacked bar chart of percent of live cells. Cell populations were identified according to the following marker combinations for each: CD4 T cells ($CD3^{+}CD19^{-}CD4^{+}$), CD8 T cells ($CD3^{+}CD19^{-}CD8a^{+}$), NK cells ($CD3^{-}CD56^{+}CD11b^{lo}$), B cells ($CD3^{-}CD56^{-}CD11b^{-}CD19^{+}$), monocytes ($CD3^{-}CD56^{-}CD11b^{hi}CD19^{-}CD66a^{-}CD14^{+}CD11c^{+}$), neutrophils ($CD3^{-}CD56^{-}CD11b^{hi}CD19^{-}CD66a^{+}HLA\text{-}DR^{lo}$). **B.** Stacked bar charts of indicated T cell subsets. **C**. Comparison of cell type frequencies between indicated platform (CyTOF or Seq-Well).

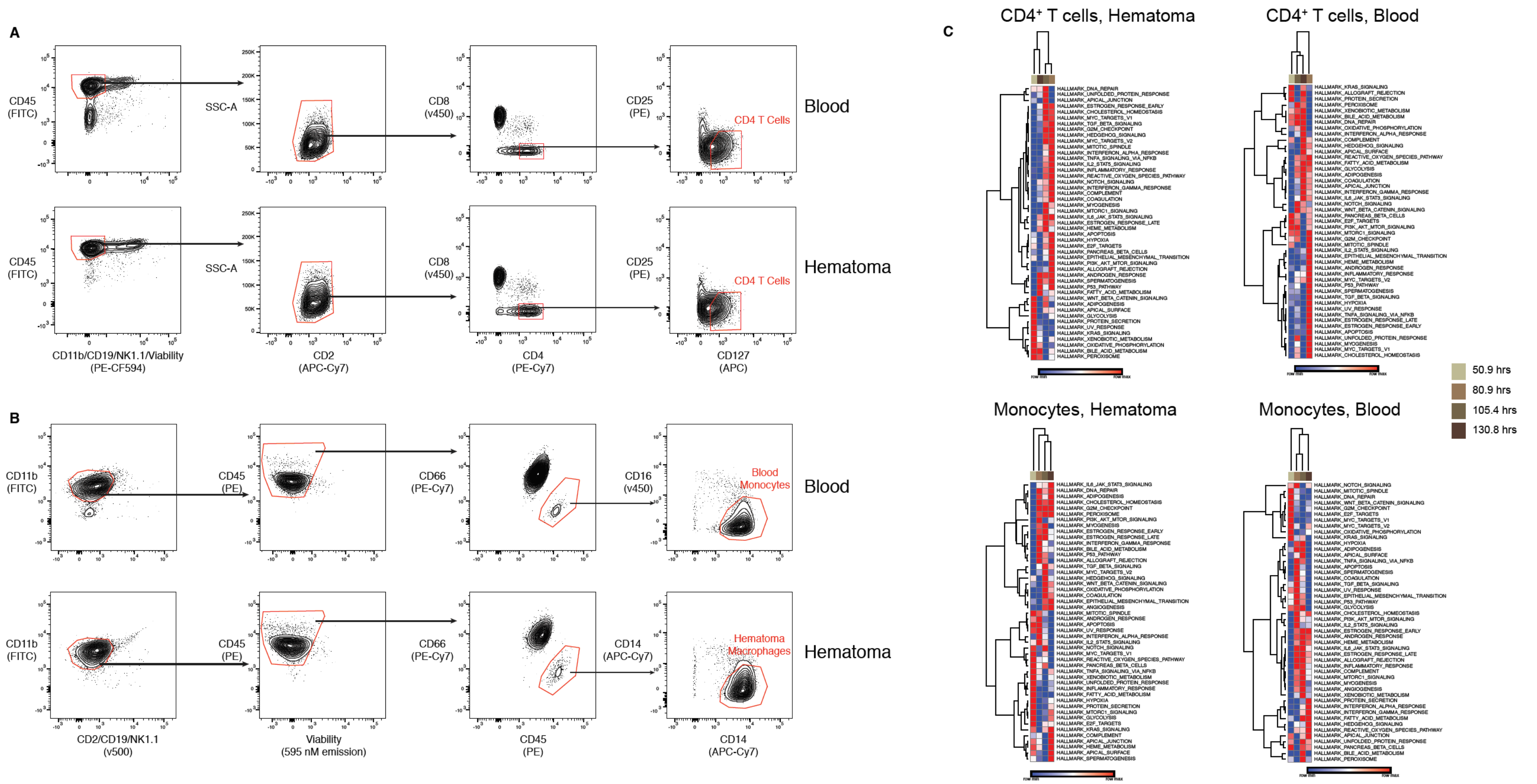

Supplemental Figure 5. Patient bulk RNA-seq. **A.** Representative sorting strategy for CD4^{+} T cells isolated from blood and hematoma for bulk RNA-seq. **B.** Representative sorting strategy for monocytes isolated from blood and hematoma for bulk RNA-seq. **C.** Heatmaps of SSGSEA results from bulk RNA-sequencing on monocytes and CD4^{+} T cells from blood or hematoma for non-overlapping time points from this patient.

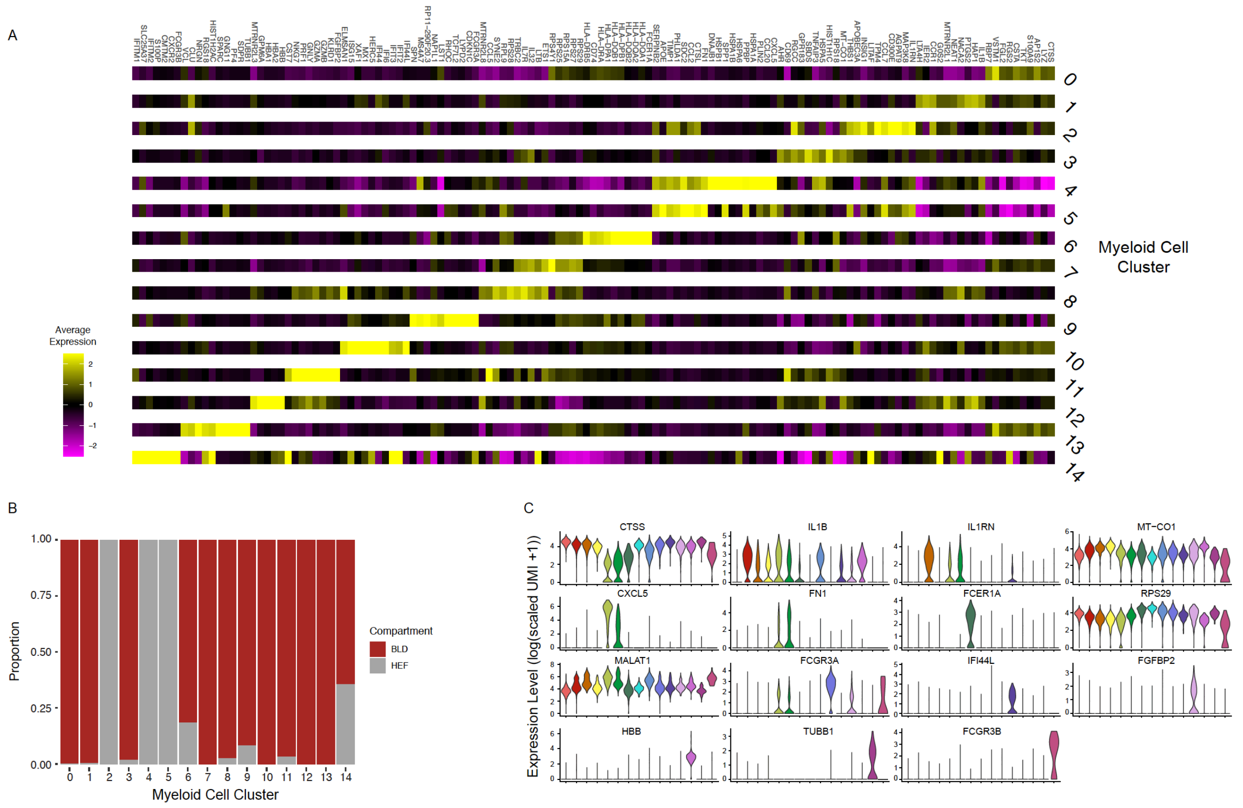

Supplemental Figure 6. Myeloid re-clustering analysis. **A**. Heatmap of top ten differentially expressed genes for each myeloid sub-cluster. **B**. Stacked bar chart showing frequency of compartment (blood or hematoma) for each myeloid sub-cluster. **C**. Violin plots of selected marker genes for each myeloid sub-cluster.

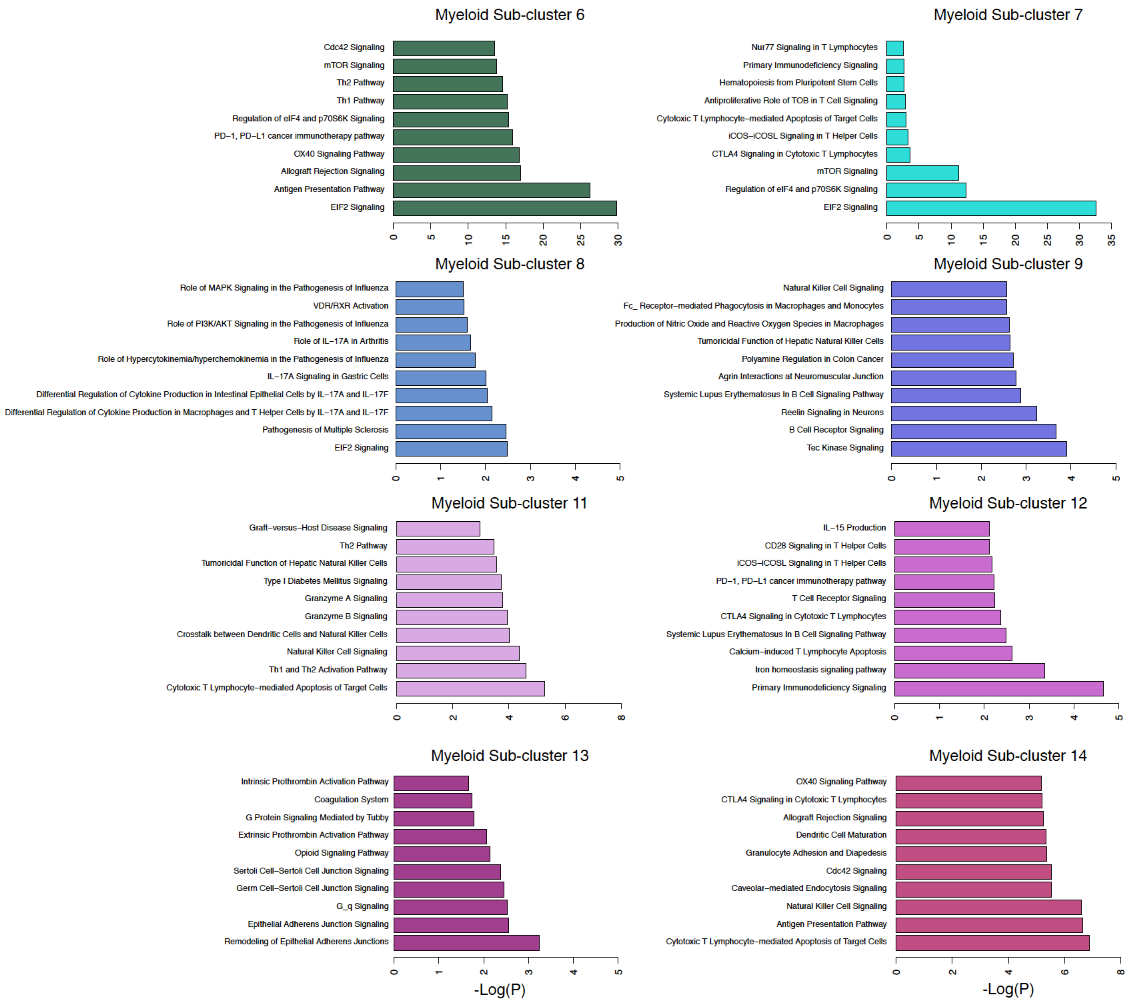

Supplemental Figure 7. Waterfall plots of top 10 enriched pathways by IPA for remaining new myeloid clusters. Results, including genes in each pathway, are also shown in Table S5.

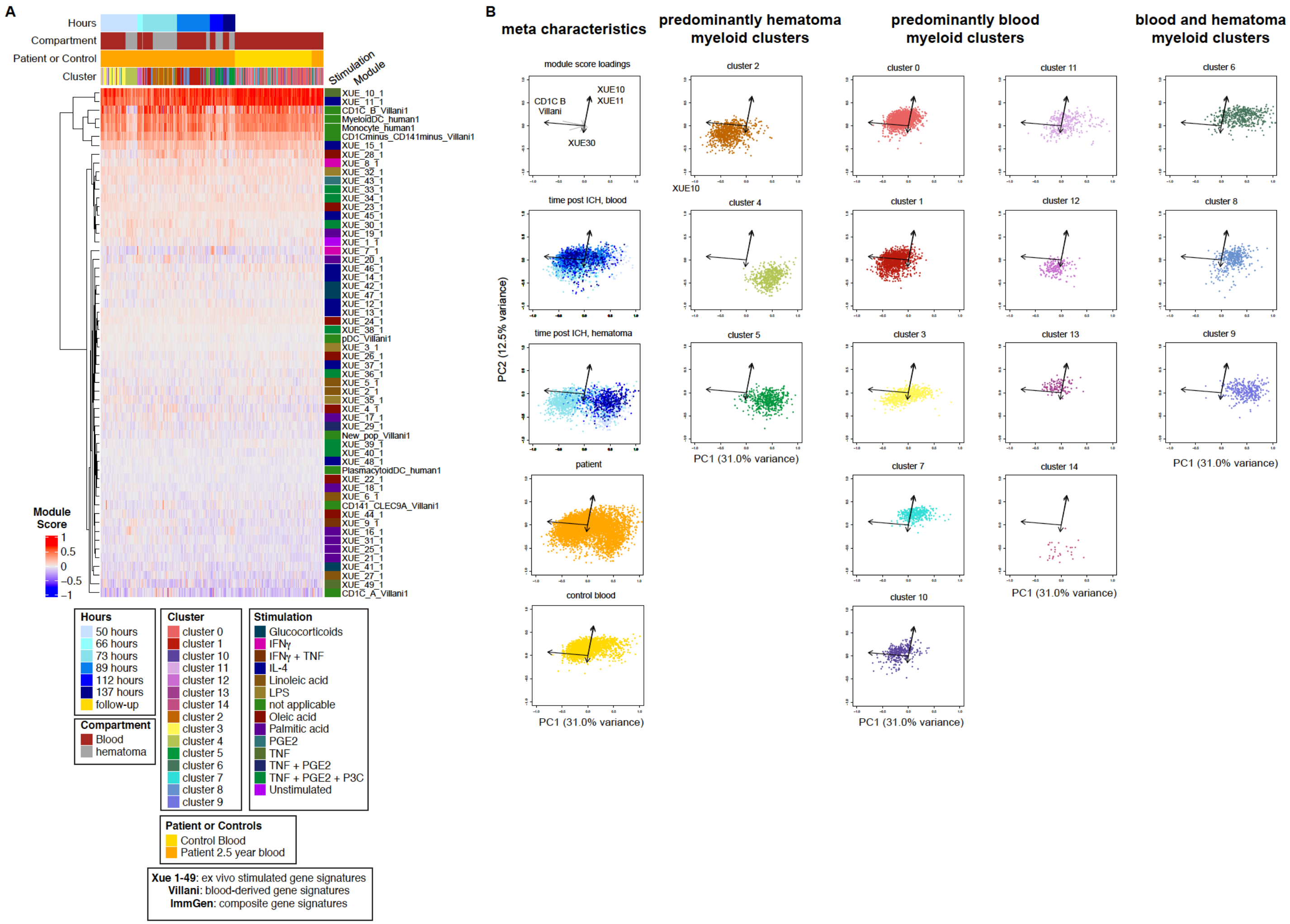

Supplemental Figure 8. **A.** Gene expression module scores were calculated for selected gene modules from the literature for each cell in our myeloid data and clustered hierarchically. The heatmap is annotated by compartment (blood or hematoma), time, source (control or patient), and myeloid sub-cluster. Each module scored over is annotated by

primary stimulation condition. B. Principal component analysis was performed on all module scores. Data are shown across the first two principal components and all module variable loadings are projected onto the top left plot and each subsequent plot shows only the top four module loading (black arrows). Data from each myeloid sub-cluster is visualized separately (sub-clusters 0-14), or as a function of time in either blood or hematoma, and as patient derived or control blood derived. Plots are grouped by metadata characteristics, predominantly blood, predominantly hematoma, or both. Gene signatures used for module scoring are described in **Table S6.**

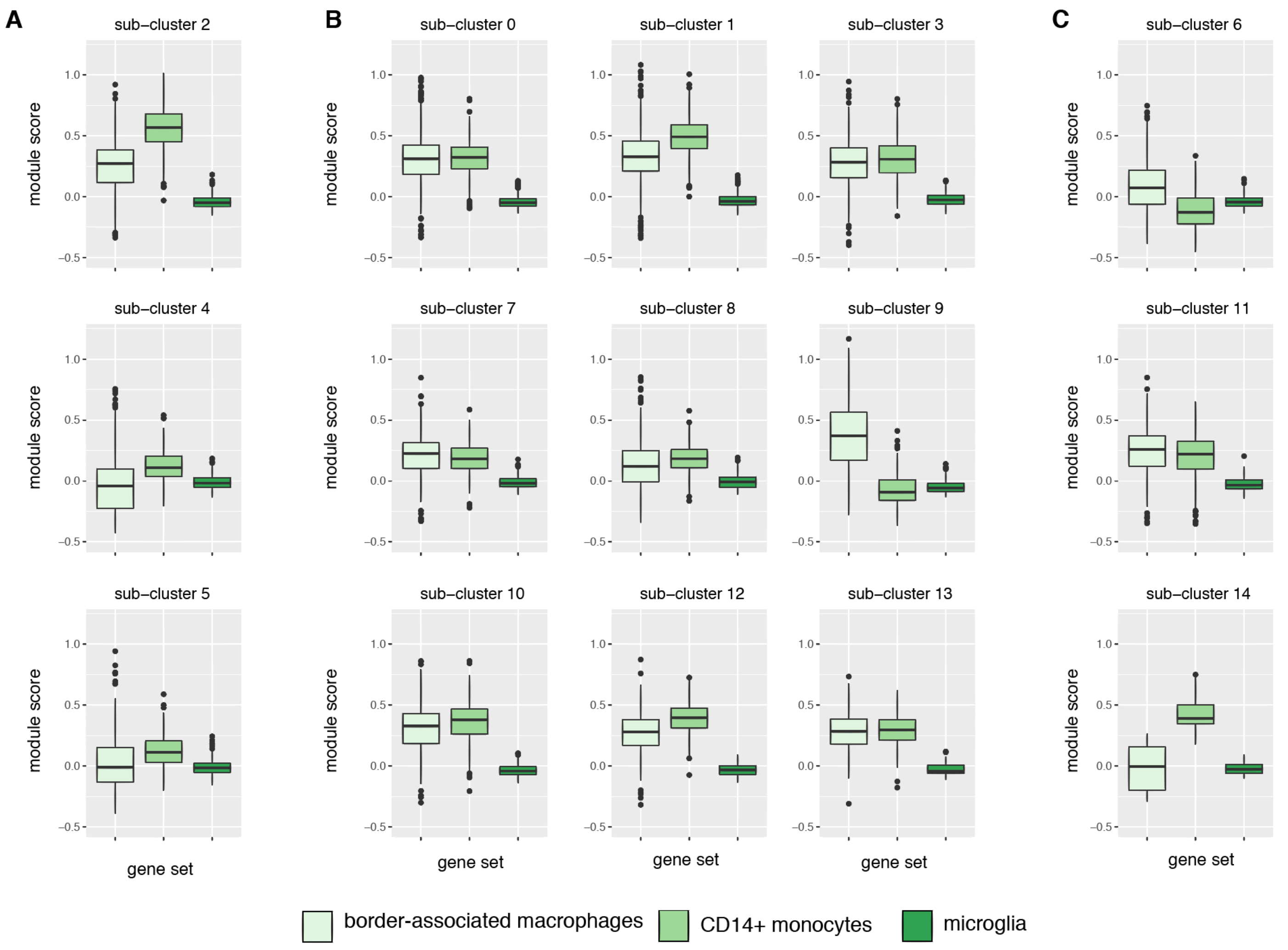

Supplemental Figure 9. Myeloid cells were scored for each indicated gene set, and box plots these scores are shown for each myeloid sub-cluster that is predominantly (A) hematoma derived, (B) blood derived, or (C) both from hematoma and blood. Gene sets are described in **Supplemental Table 6**.

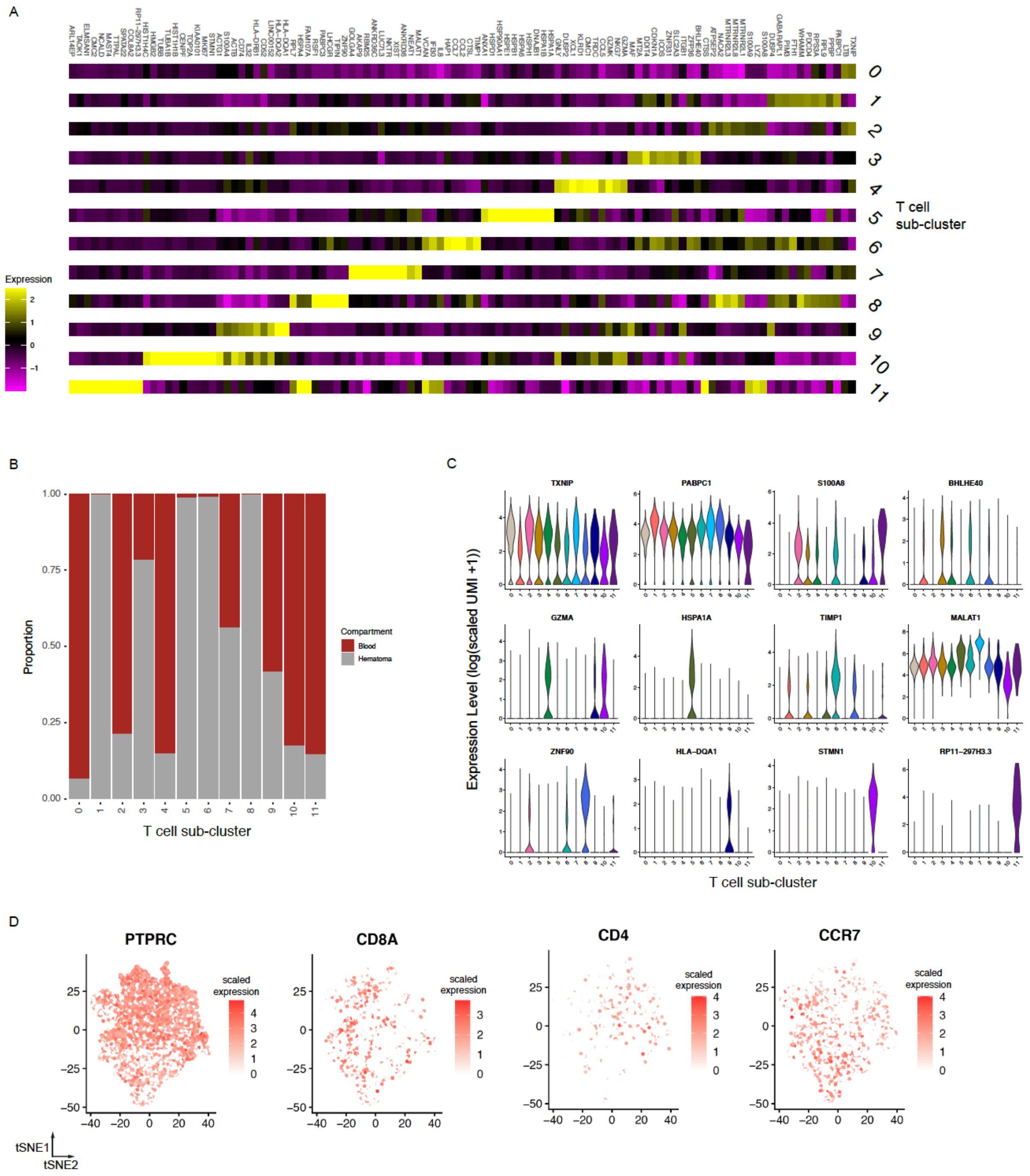

Supplemental Figure 10. T cell re-clustering analysis. **A.** Heatmap of top ten differentially expressed genes for each T cell sub-cluster. **B.** Stacked bar chart of all T cell sub-clusters by blood or hematoma. **C.** Violin plots of selected marker genes for each T cell sub-cluster. **D.** tSNEs of re-clustered T cell data colored by PTPRC, CD8A, CD4, and CCR7 expression.

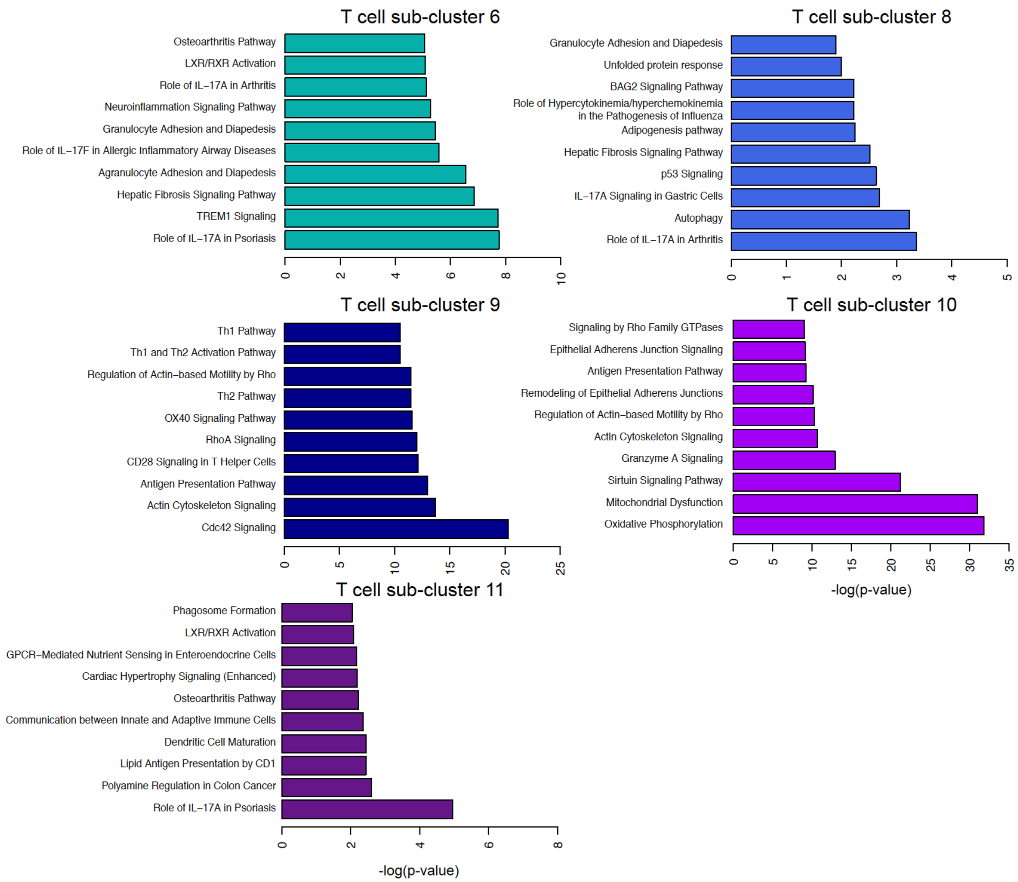

Supplemental Figure 11. Waterfall plots of top 10 enriched pathways by IPA for remaining T cell sub-clusters. Results, including genes in each pathway, are also shown in Table S9. T cell sub-cluster 0 was defined by two marker genes only, *TXNIP* and *LTB*, which precluded pathway analysis on this cluster.

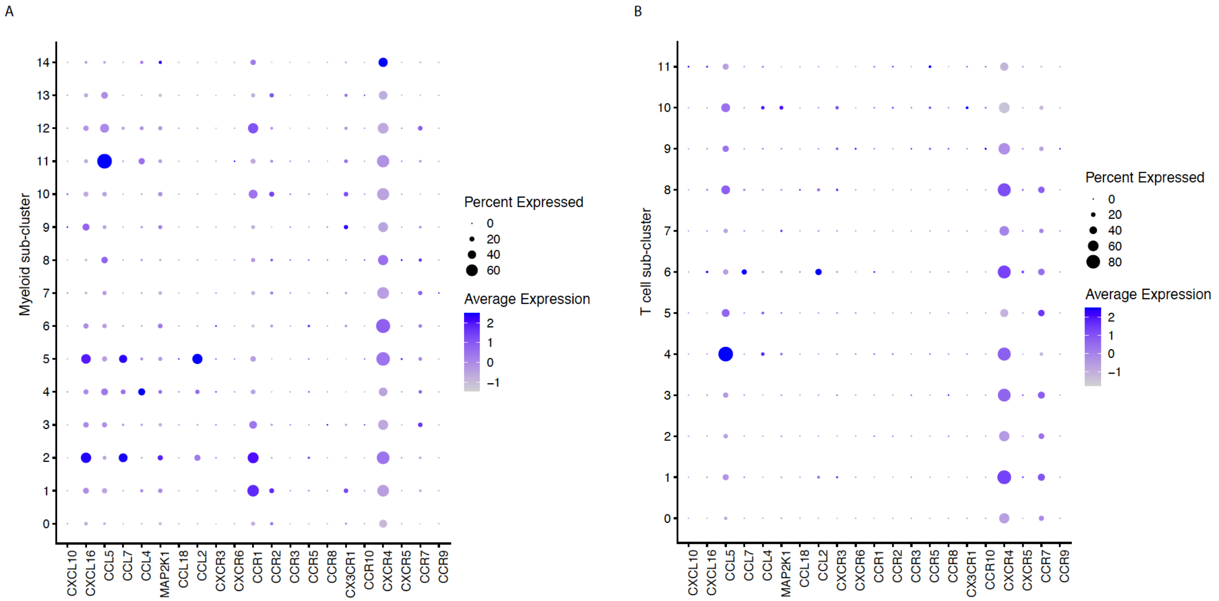

Supplemental Figure 12. Dot plots of canonical ligands and receptors for **A.** myeloid sub-clusters, and **B.** T cell sub-clusters.

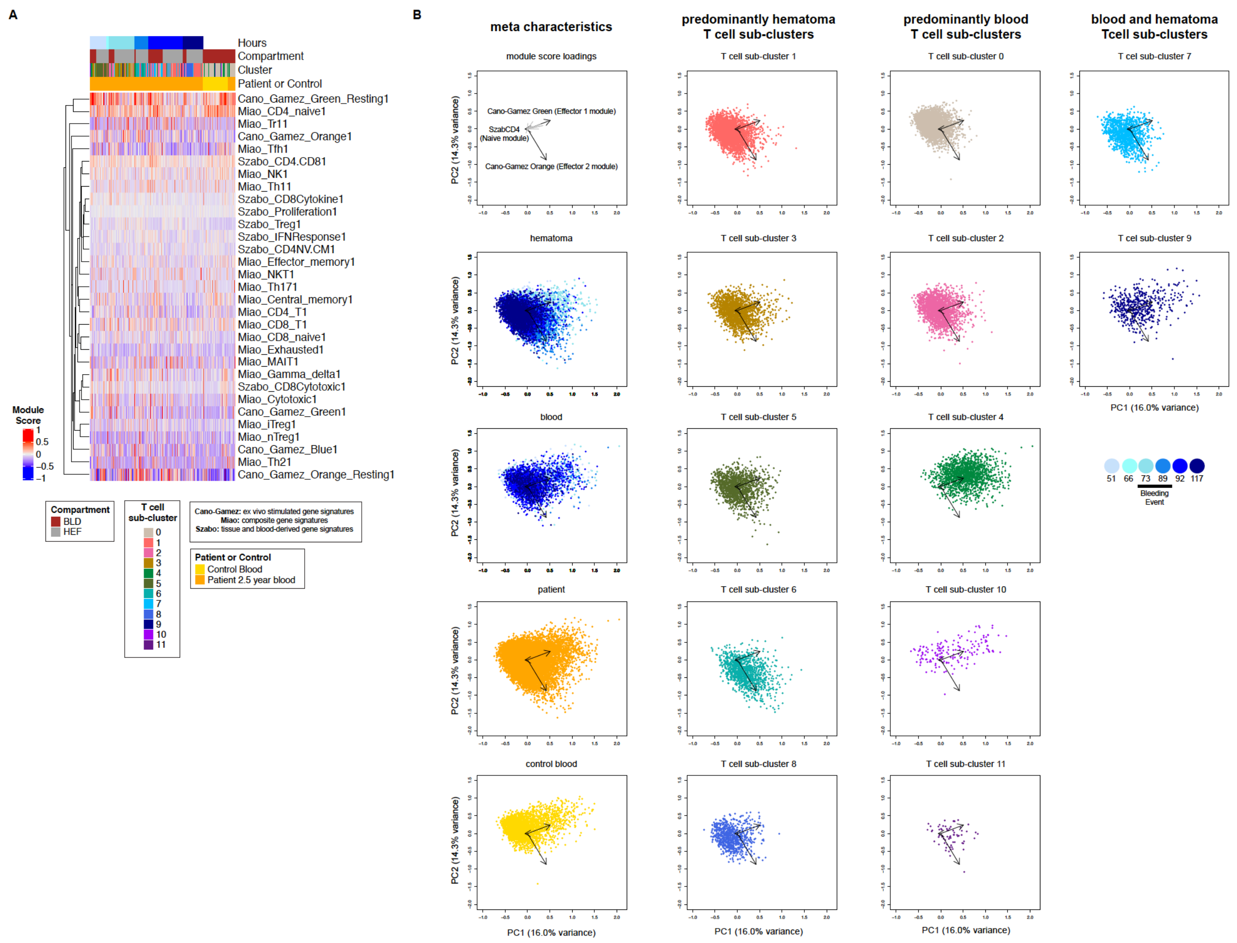

Supplemental Figure 13. A. Gene expression module scores were calculated for selected gene modules from the literature for each cell in our myeloid data and clustered hierarchically. The heatmap is annotated by compartment (blood or hematoma), time, source (control or patient), and T cell sub-cluster. **B**. Principal component analysis was performed on all module scores. Data are shown across the first two principal components and all module variable loadings are projected onto the top left plot and each subsequent plot shows only the top three module loading (black arrows). Data

from each myeloid sub-cluster is visualized separately (sub-clusters 0-11), or as a function of time in either blood or hematoma, and as patient derived or control blood derived. Plots are grouped by metadata characteristics, predominantly blood, predominantly hematoma, or both. Gene signatures used for module scoring are described in **Table S10.**

Supplemental Tables

Table S1. Patient time course.

Hrs after onset	Hematoma Volume from Catheter (mL)	Hematoma Volume on CT scan (mL)	Cell Count (x10^6)	Cells (x10^6) / mL	Sample Collection and Study Event	Surgical Event and/or Scan
0						time of onset
35.5		85.7				scan
50.9	45		2.3	0.12	SeqWell (9 mL), sort	placement of catheter and removal of liquid hematoma
52.3		36.6				scan
56.4						catheter pulled back 1.5cm
63.6		33				scan
66	11		0.4	0.09	SeqWell (4.5 mL)	sample collection followed by
						administration of tPA (dose 1)
73.2	38		4.7	0.36	SeqWell (13 mL), CyTOF	sample collection followed by
						administration of tPA (dose 2)
78.2		26.5				scan
80.9	53				Sort	administration of tPA (dose 3)
89	30		14	0.39	SeqWell (30 mL)	Sample collection followed by administration of tPA (dose 4)
93.4		25.9				scan
97.6	33		1		CyTOF	
105.4		30.2			Sort	scan
112	11		7.8	2.6	SeqWell (9 mL), CyTOF	
115		31.4				scan
121	13		2.7			
130.8		12.8			Sort	Scan

137	4		2.2	0.63	SeqWell (3.5 mL)	
140.1						Catheter removal
165.1		11.8				scan

Table S2. CyTOF antibodies.

Isotope	Epitope	Fluidigm Catalog #
89Y	CD45	3089003B
142Nd	CD19	3142001B
145Nd	CD4	3145001B
146Nd	CD8 alpha	3146001B
147Sm	CD11c	3147008B
149Sm	CD66a	3149008B
151Eu	CD14	3151009B
167Er	CD11b	3167011B
170Er	CD3	3170001B
174Yb	HLA-DR	3174001B
176Yb	CD56	3176008B
191Ir	DNA (Singlets)	201192A
193Ir	DNA (Singlets)	201192A
194Pt	Live/Dead	201194

Table S3. scRNA-seq cell counts shown in **Figure 1**.

Condition (Compartment, Time)	B cells	CD1C+ DCs	CD8 T cells	granulocytes	macrophage	monocyte/macrophage	neurons	NK cells	T cells	TOTAL
Blood, 51 hrs	33	72	251	27	20	834	0	62	483	1782
Blood, 66 hrs	18	10	0	15	13	181	0	11	57	305
Blood, 73 hrs	38	4	100	7	36	323	0	278	664	1450
Blood, 89 hrs	57	22	110	20	198	872	0	209	1543	3031
Blood, 112 hrs	49	26	147	13	40	158	0	358	1590	2381
Blood, 137 hrs	9	13	10	13	61	165	0	59	484	814
Blood, patient followup	133	88	70	20	1	327	0	197	794	1630
Blood, control 00	67	13	77	2	0	47	0	425	1184	1815
Blood, control 01	35	74	29	27	9	771	0	150	518	1613
Blood, control 02	10	60	4	15	5	1531	0	21	151	1797
Blood, control 03	56	56	192	9	0	313	0	238	756	1620
Hematoma, 51 hrs	13	2	9	1	425	0	17	18	1126	1611
Hematoma, 66 hrs	2	0	0	0	12	0	351	6	248	619
Hematoma, 73 hrs	91	55	200	20	816	39	1	278	2145	3645
Hematoma, 89 hrs	102	9	20	9	121	3	0	171	1422	1857
Hematoma, 112 hrs	83	17	12	6	235	13	8	601	2336	3311
Hematoma, 137 hrs	32	4	1	2	196	2	7	307	1890	2441

Table S4. Myeloid cell re-clustering analysis cluster membership cell counts.

Condition (Compartment, Time)	Sub-cluster 0	Sub-cluster 1	Sub-cluster 10	Sub-cluster 11	Sub-cluster 12	Sub-cluster 13	Sub-cluster 14	Sub-cluster 2	Sub-cluster 3	Sub-cluster 4	Sub-cluster 5	Sub-cluster 6	Sub-cluster 7	Sub-cluster 8	Sub-cluster 9	TOTAL
Blood, 51 hrs	11	58	3	119	1	0	1	1	612	0	0	70	6	5	39	926
Blood, 66 hrs	1	11	0	0	174	0	4	0	0	0	0	8	0	5	1	204
Blood, 73 hrs	6	290	3	9	3	0	2	0	7	0	0	4	1	32	6	363
Blood, 89 hrs	8	767	13	14	0	0	3	0	12	0	1	18	4	221	31	1092
Blood, 112 hrs	6	51	94	0	1	0	8	0	1	0	0	25	0	25	13	224
Blood, 137 hrs	10	20	120	1	1	0	2	0	0	0	0	14	0	64	7	239
Blood, patient followup	139	2	11	46	0	1	0	0	0	0	0	70	60	2	85	416
Blood, control 00	29	2	2	3	1	0	0	0	0	0	0	9	1	1	12	60
Blood, control 01	517	0	38	74	0	11	0	0	0	0	0	65	119	2	28	854
Blood, control 02	1169	0	32	31	2	84	0	0	0	0	0	50	167	5	56	1596
Blood, control 03	230	0	8	10	2	11	0	0	0	0	0	53	27	0	28	369
Hematoma, 51 hrs	0	0	0	0	0	0	0	0	0	418	7	2	0	0	0	427
Hematoma, 66 hrs	0	0	0	0	0	0	0	0	0	11	1	0	0	0	0	12
Hematoma, 73 hrs	1	3	0	8	0	0	3	734	9	57	24	56	0	4	11	910
Hematoma, 89 hrs	0	2	0	0	0	0	1	47	2	6	60	8	0	5	2	133
Hematoma, 112 hrs	0	0	0	2	0	0	5	14	1	6	207	17	0	0	13	265
Hematoma, 137 hrs	0	0	0	0	0	0	2	0	0	2	193	4	0	0	1	202

Table S5. Monocyte sub-cluster IPA results. Top 10 pathways and associated genes are shown.

myeloid sub-cluster	Ingenuity Canonical Pathways	-log(p-value)	Ratio	Molecules
myeloid subcluster 0	Retinoate Biosynthesis II	2.68	0.25	RBP7
	Pentose Phosphate Pathway (Non-oxidative Branch)	2.5	0.167	TKT
	Clathrin-mediated Endocytosis Signaling	2.35	0.0104	AP1S2,LYZ
	Pentose Phosphate Pathway	2.28	0.1	TKT
	Role of IL-17A in Psoriasis	2.16	0.0769	S100A9
	The Visual Cycle	1.98	0.05	RBP7
	Retinoate Biosynthesis I	1.75	0.0294	RBP7
	Retinol Biosynthesis	1.66	0.0238	RBP7
	PFKFB4 Signaling Pathway	1.62	0.0217	TKT
	Autophagy	1.5	0.0164	CTSS
myeloid subcluster 1	LXR/RXR Activation	5.2	0.0331	CD14,CLU,IL1B,PTGS2
	IL-10 Signaling	4.34	0.0435	CCR1,CD14,IL1B
	MIF-mediated Glucocorticoid Regulation	3.27	0.0588	CD14,PTGS2
	MIF Regulation of Innate Immunity	3.08	0.0476	CD14,PTGS2
	Role of Hypercytokinemia/hyperchemokinemia in the Pathogenesis of Influenza	3.06	0.0465	CCR1,IL1B
	Eicosanoid Signaling	2.69	0.0303	LTA4H,PTGS2
	Toll-like Receptor Signaling	2.57	0.0263	CD14,IL1B
	Sirtuin Signaling Pathway	2.52	0.0103	DUSP6,SOD2,TUBA1A
	Neuroinflammation Signaling Pathway	2.48	0.01	IL1B,PTGS2,SOD2
	Glucocorticoid Receptor Signaling	2.34	0.00893	IL1B,PTGS2,SGK1
myeloid subcluster 2	Granulocyte Adhesion and Diapedesis	11.5	0.0833	C5AR1,CCL2,CCL7,CXCL16,CXCL2,CXCL3,EZR,FPR1,IL1R1,IL1R2,IL1RAP,IL1RN,ITGA5,ITGB1,TNFRSF1A
	Agranulocyte Adhesion and Diapedesis	9.93	0.0725	ACTB,ACTG1,C5AR1,CCL2,CCL7,CXCL16,CXCL2,CXCL3,EZR,IL1R1,IL1RN,ITGA5,ITGB1,TNFRSF1A
	Osteoarthritis Pathway	7.4	0.0569	CEBPB,CTNNB1,HES1,HIF1A,IL1R1,IL1R2,IL1RAP,ITGA5,ITGB1,NAMPT,S100A8,TNFRSF1A
	Hepatic Fibrosis Signaling Pathway	6.37	0.038	CCL2,CEBPB,CTNNB1,FTH1,HIF1A,IL1R1,IL1R2,IL1RAP,IL1RN,ITGA5,ITGB1,TFRC,TIMP1,TNFRSF1A

	Caveolar-mediated Endocytosis Signaling	6.04	0.0959	ACTB,ACTG1,CD55,ITGA5,ITGAX,ITGB1,PTPN1
	TREM1 Signaling	5.96	0.0933	CCL2,CCL7,CXCL2,ITGA5,ITGAX,ITGB1,TREM1
	LXR/RXR Activation	5.59	0.0661	CCL2,CCL7,IL1R1,IL1R2,IL1RAP,IL1RN,S100A8,TNFRSF1A
	Fcγ Receptor-mediated Phagocytosis in Macrophages and Monocytes	4.23	0.0638	ACTB,ACTG1,EZR,FCGR1A,HMOX1,LCP2
	Role of Macrophages, Fibroblasts and Endothelial Cells in Rheumatoid Arthritis	4.07	0.0321	C5AR1,CCL2,CEBPB,CTNNB1,FCGR1A,IL1R1,IL1R2,IL1RAP,IL1RN ,TNFRSF1A
	Leukocyte Extravasation Signaling	4.06	0.0406	ACTB,ACTG1,CD44,CTNNB1,EZR,ITGA5,ITGB1,TIMP1
myeloid subcluster 3	Granzyme A Signaling	6.06	0.158	H1-2,H1-3,H1-4
	Glucocorticoid Receptor Signaling	3.48	0.0119	ANXA1,CCNH,HSPA5,TSC22D3
	Toll-like Receptor Signaling	2.57	0.0263	TNFAIP3,UBC
	Sirtuin Signaling Pathway	2.52	0.0103	H1-2,H1-3,H1-4
	NER Pathway	2.32	0.0194	CCNH,H4C3
	Trans, trans-farnesyl Diphosphate Biosynthesis	2.3	0.2	IDI1
	Protein Kinase A Signaling	2.14	0.00754	H1-2,H1-3,H1-4
	Necroptosis Signaling Pathway	1.96	0.0127	PELI1,UBC
	NF-κB Signaling	1.85	0.0112	PELI1,TNFAIP3
	Mevalonate Pathway I	1.85	0.0714	IDI1
myeloid subcluster 4	Glycolysis I	13	0.346	ALDOA,ENO1,ENO2,GAPDH,GPI,PFKP,PGK1,PKM,TPI1
	Aldosterone Signaling in Epithelial Cells	11.1	0.0886	DNAJA1,DNAJB1,HSP90AA1,HSP90AB1,HSP90B1,HSPA1A/HSPA1 B,HSPA6,HSPA8,HSPB1,HSPD1,HSPE1,HSPH1,PDIA3,PIK3CB
	Protein Ubiquitination Pathway	7.97	0.0513	DNAJA1,DNAJB1,HSP90AA1,HSP90AB1,HSP90B1,HSPA1A/HSPA1 B,HSPA6,HSPA8,HSPB1,HSPD1,HSPE1,HSPH1,PSMA6,UBB
	Role of IL-17A in Psoriasis	7.67	0.385	CCL20,CXCL1,CXCL5,CXCL6,CXCL8
	Gluconeogenesis I	7.61	0.231	ALDOA,ENO1,ENO2,GAPDH,GPI,PGK1
	HIF1α Signaling	6.92	0.0796	EGLN3,HSP90AA1,JUN,LDHA,MMP19,PIK3CB,RALA,SLC2A1,VEGF A
	Role of IL-17A in Arthritis	6.9	0.127	CCL20,CXCL1,CXCL5,CXCL6,CXCL8,NFKBIA,PIK3CB
	Unfolded protein response	6.84	0.125	CD82,HSP90B1,HSPA1A/HSPA1B,HSPA6,HSPA8,HSPH1,PDIA6
	Role of PKR in Interferon Induction and Antiviral Response	6.79	0.0769	HSP90AA1,HSP90AB1,HSP90B1,HSPA1A/HSPA1B,HSPA6,HSPA8, JUN,MARCO,NFKBIA
	Granulocyte Adhesion and Diapedesis	6.16	0.0556	CCL20,CCL3,CCL4,CXCL1,CXCL5,CXCL6,CXCL8,MMP19,PPBP,SD C2
myeloid subcluster 5	Senescence Pathway	3.96	0.0291	FOXO3,HIPK2,MAP2K3,MAPKAPK2,PIK3R5,SERPINE1,SMAD7,TB K1
	Phagosome Maturation	3.82	0.0397	ATP6V0B,CALR,CTSB,CTSV,RAB5C,TUBA1C

	FGF Signaling	2.99	0.0476	ATF4,MAP2K3,MAPKAPK2,PIK3R5
	Death Receptor Signaling	2.86	0.044	LMNA,TBK1,TIPARP,TNFRSF21
	PFKFB4 Signaling Pathway	2.73	0.0652	ATF4,FBP1,MAP2K3
	Type I Diabetes Mellitus Signaling	2.54	0.036	CD86,IFNGR2,IRAK1,MAP2K3
	Role of IL-17A in Arthritis	2.51	0.0545	MAP2K3,MAPKAPK2,PIK3R5
	p38 MAPK Signaling	2.45	0.0339	ATF4,IRAK1,MAP2K3,MAPKAPK2
	Clathrin-mediated Endocytosis Signaling	2.44	0.0259	APOC1,APOE,DAB2,PIK3R5,RAB5C
	Autophagy	2.38	0.0492	CTSB,CTSV,RB1CC1
myeloid subcluster 6	EIF2 Signaling	29.8	0.103	RPL10A,RPL13A,RPL17,RPL22,RPL23A,RPL27A,RPL3,RPL35,RPL5,RPLP0,RPS11,RPS2,RPS21,RPS23,RPS24,RPS29,RPS3A,RPS4X,RPS5,RPS6,RPS7,RPS8,RPSA
	Antigen Presentation Pathway	26.3	0.359	CD74,CIITA,HLA-DMA,HLA-DMB,HLA-DOA,HLA-DPA1,HLA-DPB1,HLA-DQA1,HLA-DQA2,HLA-DQB1,HLA-DQB2,HLA-DRA,HLA-DRB1,HLA-DRB5
	Allograft Rejection Signaling	17	0.14	HLA-DMA,HLA-DMB,HLA-DOA,HLA-DPA1,HLA-DPB1,HLA-DQA1,HLA-DQA2,HLA-DQB1,HLA-DQB2,HLA-DRA,HLA-DRB1,HLA-DRB5
	OX40 Signaling Pathway	16.8	0.133	HLA-DMA,HLA-DMB,HLA-DOA,HLA-DPA1,HLA-DPB1,HLA-DQA1,HLA-DQA2,HLA-DQB1,HLA-DQB2,HLA-DRA,HLA-DRB1,HLA-DRB5
	PD-1, PD-L1 cancer immunotherapy pathway	15.9	0.113	HLA-DMA,HLA-DMB,HLA-DOA,HLA-DPA1,HLA-DPB1,HLA-DQA1,HLA-DQA2,HLA-DQB1,HLA-DQB2,HLA-DRA,HLA-DRB1,HLA-DRB5
	Regulation of eIF4 and p70S6K Signaling	15.4	0.0828	RPS11,RPS2,RPS21,RPS23,RPS24,RPS29,RPS3A,RPS4X,RPS5,RPS6,RPS7,RPS8,RPSA
	Th1 Pathway	15.2	0.0992	HLA-DMA,HLA-DMB,HLA-DOA,HLA-DPA1,HLA-DPB1,HLA-DQA1,HLA-DQA2,HLA-DQB1,HLA-DQB2,HLA-DRA,HLA-DRB1,HLA-DRB5
	Th2 Pathway	14.6	0.0882	HLA-DMA,HLA-DMB,HLA-DOA,HLA-DPA1,HLA-DPB1,HLA-DQA1,HLA-DQA2,HLA-DQB1,HLA-DQB2,HLA-DRA,HLA-DRB1,HLA-DRB5
	mTOR Signaling	13.8	0.0619	RPS11,RPS2,RPS21,RPS23,RPS24,RPS29,RPS3A,RPS4X,RPS5,RPS6,RPS7,RPS8,RPSA
	Cdc42 Signaling	13.5	0.0719	HLA-DMA,HLA-DMB,HLA-DOA,HLA-DPA1,HLA-DPB1,HLA-DQA1,HLA-DQA2,HLA-DQB1,HLA-DQB2,HLA-DRA,HLA-DRB1,HLA-DRB5
myeloid subcluster 7	EIF2 Signaling	32.6	0.0893	RPL13,RPL14,RPL27,RPL30,RPL31,RPL34,RPL35A,RPL38,RPL39,RPL4,RPL41,RPS10,RPS12,RPS15A,RPS20,RPS25,RPS27,RPS27A,RPS3,RPS4Y1
	Regulation of eIF4 and p70S6K Signaling	12.3	0.0573	RPS10,RPS12,RPS15A,RPS20,RPS25,RPS27,RPS27A,RPS3,RPS4Y1
	mTOR Signaling	11.2	0.0429	RPS10,RPS12,RPS15A,RPS20,RPS25,RPS27,RPS27A,RPS3,RPS4Y1
	CTLA4 Signaling in Cytotoxic T Lymphocytes	3.62	0.0337	CD3D,CD3G,TRAT1

	iCOS-iCOSL Signaling in T Helper Cells	3.34	0.027	CD3D,CD3G,TRAT1
	Cytotoxic T Lymphocyte-mediated Apoptosis of Target Cells	3.01	0.0588	CD3D,CD3G
	Antiproliferative Role of TOB in T Cell Signaling	2.89	0.0513	CD3D,CD3G
	Hematopoiesis from Pluripotent Stem Cells	2.69	0.0408	CD3D,CD3G
	Primary Immunodeficiency Signaling	2.67	0.04	CD3D,IL7R
	Nur77 Signaling in T Lymphocytes	2.53	0.0339	CD3D,CD3G
myeloid subcluster 8	EIF2 Signaling	2.48	0.00893	RPLP2,RPS28
	Pathogenesis of Multiple Sclerosis	2.45	0.111	CCL5
	Differential Regulation of Cytokine Production in Macrophages and T Helper Cells by IL-17A and IL-17F	2.15	0.0556	CCL5
	Differential Regulation of Cytokine Production in Intestinal Epithelial Cells by IL-17A and IL-17F	2.04	0.0435	CCL5
	IL-17A Signaling in Gastric Cells	2.01	0.04	CCL5
	Role of Hypercytokinemia/hyperchemokinemia in the Pathogenesis of Influenza	1.77	0.0233	CCL5
	Role of IL-17A in Arthritis	1.67	0.0182	CCL5
	Role of PI3K/AKT Signaling in the Pathogenesis of Influenza	1.6	0.0156	CCL5
	VDR/RXR Activation	1.52	0.0128	CCL5
	Role of MAPK Signaling in the Pathogenesis of Influenza	1.5	0.0125	CCL5
myeloid subcluster 9	Tec Kinase Signaling	3.91	0.0305	GNG2,HCK,ITGA4,LYN,RHOC
	B Cell Receptor Signaling	3.66	0.027	LYN,PAG1,PIK3AP1,POU2F2,PTPN6
	Reelin Signaling in Neurons	3.24	0.031	HCK,ITGA4,ITGAL,LYN
	Systemic Lupus Erythematosus In B Cell Signaling Pathway	2.88	0.0182	HCK,LYN,PAG1,PIK3AP1,PTPN6
	Agrin Interactions at Neuromuscular Junction	2.78	0.038	ITGA4,ITGAL,UTRN
	Polyamine Regulation in Colon Cancer	2.72	0.0909	PSME2,SAT1
	Tumoricidal Function of Hepatic Natural Killer Cells	2.64	0.0833	BID,ITGAL
	Production of Nitric Oxide and Reactive Oxygen Species in Macrophages	2.63	0.0213	PTPN6,RHOC,SERPINA1,TNFRSF1B
	Fcγ Receptor-mediated Phagocytosis in Macrophages and Monocytes	2.57	0.0319	FCGR3A/FCGR3B,HCK,LYN
	Natural Killer Cell Signaling	2.56	0.0203	FCGR3A/FCGR3B,ITGAL,LILRB1,PTPN6

myeloid subcluster 10	Interferon Signaling	12.8	0.194	IFI6,IFIT1,IFIT3,ISG15,MX1,OAS1,STAT1
	Systemic Lupus Erythematosus In B Cell Signaling Pathway	6.49	0.0255	FOS,IFIT2,IFIT3,ISG15,STAT1,TNFSF10,TNFSF13B
	Role of Pattern Recognition Receptors in Recognition of Bacteria and Viruses	5.25	0.0325	EIF2AK2,OAS1,OAS2,TNFSF10,TNFSF13B
	UVA-Induced MAPK Signaling	4.67	0.0408	FOS,PARP14,PARP9,STAT1
	Role of PKR in Interferon Induction and Antiviral Response	4.37	0.0342	EIF2AK2,FOS,HSPA4,STAT1
	Inhibition of ARE-Mediated mRNA Degradation Pathway	4.29	0.0328	TNFSF10,TNFSF13B,ZFP36,ZFP36L2
	Retinoic acid Mediated Apoptosis Signaling	3.86	0.05	PARP14,PARP9,TNFSF10
	Activation of IRF by Cytosolic Pattern Recognition Receptors	3.8	0.0476	IFIT2,ISG15,STAT1
	PDGF Signaling	3.4	0.0349	EIF2AK2,FOS,STAT1
	Death Receptor Signaling	3.32	0.033	PARP14,PARP9,TNFSF10
myeloid subcluster 11	Cytotoxic T Lymphocyte-mediated Apoptosis of Target Cells	5.27	0.0882	CD247,GZMB,PRF1
	Th1 and Th2 Activation Pathway	4.61	0.0234	CD247,IL2RB,JUN,KLRD1
	Natural Killer Cell Signaling	4.37	0.0203	CD247,IL2RB,KLRB1,KLRD1
	Crosstalk between Dendritic Cells and Natural Killer Cells	4.01	0.0337	IL2RB,KLRD1,PRF1
	Granzyme B Signaling	3.93	0.125	GZMB,PRF1
	Granzyme A Signaling	3.78	0.105	GZMA,PRF1
	Type I Diabetes Mellitus Signaling	3.73	0.027	CD247,GZMB,PRF1
	Tumoricidal Function of Hepatic Natural Killer Cells	3.57	0.0833	GZMB,PRF1
	Th2 Pathway	3.47	0.0221	CD247,IL2RB,JUN
	Graft-versus-Host Disease Signaling	2.97	0.0417	GZMB,PRF1
myeloid subcluster 12	Primary Immunodeficiency Signaling	4.65	0.06	IGHA1,LCK,ZAP70
	Iron homeostasis signaling pathway	3.35	0.0219	CIAO2B,HBA1/HBA2,HBB
	Calcium-induced T Lymphocyte Apoptosis	2.62	0.0303	LCK,ZAP70
	Systemic Lupus Erythematosus In B Cell Signaling Pathway	2.48	0.0109	IGHA1,ISG20,LCK
	CTLA4 Signaling in Cytotoxic T Lymphocytes	2.37	0.0225	LCK,ZAP70
	T Cell Receptor Signaling	2.23	0.019	LCK,ZAP70
	PD-1, PD-L1 cancer immunotherapy pathway	2.22	0.0189	LCK,ZAP70
	iCOS-iCOSL Signaling in T Helper Cells	2.18	0.018	LCK,ZAP70

	CD28 Signaling in T Helper Cells	2.12	0.0167	LCK,ZAP70
	IL-15 Production	2.11	0.0165	LCK,ZAP70
myeloid subcluster 13	Remodeling of Epithelial Adherens Junctions	3.24	0.0294	TUBB1,VCL
	Epithelial Adherens Junction Signaling	2.55	0.0132	TUBB1,VCL
	Gαq Signaling	2.52	0.0127	GNG11,RGS18
	Germ Cell-Sertoli Cell Junction Signaling	2.45	0.0117	TUBB1,VCL
	Sertoli Cell-Sertoli Cell Junction Signaling	2.38	0.0108	TUBB1,VCL
	Opioid Signaling Pathway	2.14	0.0081	GNG11,RGS18
	Extrinsic Prothrombin Activation Pathway	2.07	0.0625	F13A1
	G Protein Signaling Mediated by Tubby	1.79	0.0323	GNG11
	Coagulation System	1.74	0.0286	F13A1
	Intrinsic Prothrombin Activation Pathway	1.66	0.0238	F13A1
myeloid subcluster 14	Cytotoxic T Lymphocyte-mediated Apoptosis of Target Cells	6.89	0.118	B2M,HLA-B,HLA-C,HLA-E
	Antigen Presentation Pathway	6.65	0.103	B2M,HLA-B,HLA-C,HLA-E
	Natural Killer Cell Signaling	6.61	0.0305	B2M,FCGR3A/FCGR3B,HLA-B,HLA-C,HLA-E,LIMK2
	Caveolar-mediated Endocytosis Signaling	5.54	0.0548	B2M,HLA-B,HLA-C,HLA-E
	Cdc42 Signaling	5.53	0.0299	B2M,HLA-B,HLA-C,HLA-E,LIMK2
	Granulocyte Adhesion and Diapedesis	5.37	0.0278	CSF3R,CXCR2,FPR2,MMP25,SELL
	Dendritic Cell Maturation	5.34	0.0273	B2M,FCGR3A/FCGR3B,HLA-B,HLA-C,HLA-E
	Allograft Rejection Signaling	5.25	0.0465	B2M,HLA-B,HLA-C,HLA-E
	CTLA4 Signaling in Cytotoxic T Lymphocytes	5.19	0.0449	B2M,HLA-B,HLA-C,HLA-E
	OX40 Signaling Pathway	5.17	0.0444	B2M,HLA-B,HLA-C,HLA-E

Table S6. Human monocyte gene sets used for module scoring.

Gene Set Name	Description	Reference
CD141_CLEC9A_Villani	Generated from unstimulated human PBMCs, identified by scRNA-seq.	Villani et al.(1)
CD1C_A_Villani	Generated from unstimulated human PBMCs, identified by scRNA-seq.	Villani et al.(1)
CD1C_B_Villani	Generated from unstimulated human PBMCs, identified by scRNA-seq.	Villani et al.(1)
CD1Cminus_CD141minus_Villani	Generated from unstimulated human PBMCs, identified by scRNA-seq.	Villani et al.(1)
New_pop_Villani	Generated from unstimulated human PBMCs, identified by scRNA-seq.	Villani et al.(1)
pDC_Villani	Generated from unstimulated human PBMCs, identified by scRNA-seq.	Villani et al.(1)
XUE_Module1 to XUE_Module49	Generated by comparative/ integrative analysis on several microarray datasets.	Xue et al.(1)
Monocyte_human	ImmGenn database	Shay et al.(2)
MyeloidDC_human	ImmGenn database	Shay et al.(2)
PlasmacytoidDC_human	ImmGenn database	Shay et al.(2)

CD14_Monocytes_blood	Generated from unstimulated human PBMCs, identified by scRNA-seq.	Villani et al.(1)
Microglia	Transcriptional profiling of human microglia and resident macropahges; gene expression in tissue macropahges in mice.	Poel et al., Gautier et al., and Gosselin et al. (3-5)
BAMs	Single cell atlas of mouse brain macrophages and fate mapping in mice.	Van Hove et al., and Goldmann et al. (6, 7)

Table S7. Wilcox rank sum pairwise test results for CD1CB ($CD14^+$ monocyte) signature module scores, data presented in Figure 2. Table displays adjusted p values (p_{adj}) for pairwise comparisons for each indicated myeloid sub-cluster. P values are colored as follows: blue ($0.01 \leq p_{adj} < 0.05$, *), purple ($0.001 \leq p_{adj} < 0.01$, **), white ($0 \leq p_{adj} < 0.001$, ***), gray (not significant, padj < 0.05).

Sub-cluster	0	1	2	3	4	5	6	7	8	9	10	11	12	13
**1**	1.68E-47	-	-	-	-	-	-	-	-	-	-	-	-	-
**2**	6.19E-71	3.28E-10	-	-	-	-	-	-	-	-	-	-	-	-
**3**	1.15E-16	1.72E-57	1.66E-69	-	-	-	-	-	-	-	-	-	-	-
**4**	3.08E-249	3.56E-225	1.74E-193	1.74E-152	-	-	-	-	-	-	-	-	-	-
**5**	2.06E-224	2.56E-212	3.16E-183	5.98E-125	2.91E-08	-	-	-	-	-	-	-	-	-
**6**	2.95E-187	7.73E-189	1.50E-166	6.25E-98	2.16E-06	6.31E-01	-	-	-	-	-	-	-	-
**7**	1.03E-65	8.67E-103	2.51E-100	1.06E-14	4.04E-106	7.82E-78	2.00E-54	-	-	-	-	-	-	-
**8**	6.51E-92	6.62E-120	4.97E-112	2.32E-30	3.11E-92	1.19E-60	3.87E-38	4.82E-06	-	-	-	-	-	-
**9**	4.48E-184	3.41E-169	1.08E-151	9.39E-134	3.03E-38	1.19E-52	1.00E-37	1.35E-104	1.01E-98	-	-	-	-	-
**10**	1.29E-02	6.82E-24	1.02E-35	5.30E-04	1.66E-117	1.91E-103	4.21E-86	6.68E-26	3.08E-41	3.85E-104	-	-	-	-
**11**	1.80E-39	2.42E-71	4.43E-75	5.83E-09	1.75E-72	6.97E-55	3.53E-41	3.48E-01	3.85E-06	1.62E-78	1.29E-16	-	-	-
**12**	2.78E-02	1.59E-17	4.05E-26	3.21E-03	1.69E-83	1.16E-74	9.99E-63	6.67E-21	1.16E-32	1.11E-76	9.52E-01	1.63E-13	-	-
**13**	7.50E-04	4.59E-15	1.94E-20	4.75E-01	2.89E-52	1.81E-45	6.63E-37	5.65E-09	9.59E-17	1.69E-51	1.14E-01	6.67E-06	1.23E-01	-
**14**	4.48E-08	1.68E-12	2.35E-13	1.57E-03	1.35E-14	1.28E-10	1.04E-07	4.92E-01	3.47E-01	1.00E-17	4.53E-06	3.72E-01	3.39E-06	3.69E-04

Table S8. T cell sub-clustering analysis cluster membership cell counts. Sub-clusters are indicated by NT0-NT11.

Condition (Compartment, Time)	NT 0	NT 1	NT 2	NT 3	NT 4	NT 5	NT 6	NT 7	NT 8	NT 9	NT 10	NT 11	Total
Blood, 51 hrs	34	1	10	323	268	17	0	1	0	52	15	0	721
Blood, 66 hrs	1	0	17	0	3	0	3	4	0	0	0	0	28
Blood, 73 hrs	27	0	485	21	118	0	0	14	0	22	8	0	695
Blood, 89 hrs	3	0	49	1	101	0	1	8	0	10	11	0	184
Blood, 112 hrs	326	0	579	8	237	0	3	456	0	55	12	5	1681
Blood, 137 hrs	4	0	365	0	14	0	6	22	1	2	0	53	467
Blood, patient followup	645	0	6	6	95	0	0	0	0	58	48	0	858
Blood, control 00	1069	1	1	16	109	0	0	2	0	45	17	0	1260
Blood, control 01	424	0	4	1	61	0	0	0	0	18	15	0	523
Blood, control 02	133	0	2	3	5	0	0	0	0	3	3	0	149
Blood, control 03	555	1	11	6	325	0	0	4	0	29	8	0	939
Hematoma, 51 hrs	4	4	2	18	8	1087	1	11	0	0	0	0	1135
Hematoma, 66 hrs	3	7	3	1	0	217	4	11	2	0	0	0	248
Hematoma, 73 hrs	194	37	345	1266	190	19	81	56	0	133	11	0	2332
Hematoma, 89 hrs	2	116	54	66	23	4	1014	122	4	15	4	0	1424
Hematoma, 112 hrs	25	1774	6	45	10	1	43	355	18	54	12	5	2348
Hematoma, 137 hrs	2	1064	2	0	0	5	12	98	692	9	2	5	1891

Table S9. T cell sub-cluster IPA results. Top 10 pathways and associated genes are shown.

T cell sub-cluster	Ingenuity Canonical Pathways	-log(p-value)	Ratio	Molecules
T cell subcluster 1	EIF2 Signaling	3.16	0.0134	PABPC1,RPL9,RPS3A
	Regulation of eIF4 and p70S6K Signaling	2.17	0.0127	PABPC1,RPS3A
	Antiproliferative Role of TOB in T Cell Signaling	1.52	0.0256	PABPC1
	Hepatic Fibrosis Signaling Pathway	1.47	0.00543	FTH1,PDCD4
	Transcriptional Regulatory Network in Embryonic Stem Cells	1.38	0.0185	SKIL
	SAPK/JNK Signaling	1.11	0.0098	DUSP4
	PD-1, PD-L1 cancer immunotherapy pathway	1.09	0.00943	PDCD4
	Inhibition of ARE-Mediated mRNA Degradation Pathway	1.03	0.0082	CNOT1
	Iron homeostasis signaling pathway	0.987	0.0073	FTH1
	Granulocyte Adhesion and Diapedesis	0.873	0.00556	PPBP
T cell subcluster 2	Role of IL-17A in Psoriasis	5.2	0.154	S100A8,S100A9
	LXR/RXR Activation	3.24	0.0165	LYZ,S100A8
	Atherosclerosis Signaling	3.2	0.0159	LYZ,S100A8
	IL-12 Signaling and Production in Macrophages	3.16	0.0152	LYZ,S100A8
	Production of Nitric Oxide and Reactive Oxygen Species in Macrophages	2.86	0.0106	LYZ,S100A8
	Clathrin-mediated Endocytosis Signaling	2.83	0.0104	LYZ,S100A8
	Osteoarthritis Pathway	2.76	0.00948	S100A8,S100A9
	Autophagy	1.73	0.0164	CTSS
	Adipogenesis pathway	1.39	0.00746	TXNIP
	Phagosome Maturation	1.34	0.00662	CTSS
T cell subcluster 3	GADD45 Signaling	3.86	0.105	CDKN1A,GADD45A
	HGF Signaling	3.85	0.027	CDKN1A,FOS,ITGB1
	Systemic Lupus Erythematosus In T Cell Signaling Pathway	3.65	0.012	CREM,FOS,GADD45A,ICOS
	TNFR2 Signaling	3.46	0.0667	FOS,TNFAIP3
	Cell Cycle: G2/M DNA Damage Checkpoint Regulation	3.03	0.0408	CDKN1A,GADD45A

	TNFR1 Signaling	3.01	0.04	FOS,TNFAIP3
	CD40 Signaling	2.79	0.0308	FOS,TNFAIP3
	Toll-like Receptor Signaling	2.65	0.0263	FOS,TNFAIP3
	VDR/RXR Activation	2.63	0.0256	CDKN1A,GADD45A
	Role of BRCA1 in DNA Damage Response	2.61	0.025	CDKN1A,GADD45A
T cell subcluster 4	IL-17A Signaling in Gastric Cells	5.28	0.12	CCL5,FOS,JUN
	Chemokine Signaling	3.75	0.0375	CCL5,FOS,JUN
	Granzyme B Signaling	3.67	0.125	GZMB,PRF1
	CCR5 Signaling in Macrophages	3.55	0.0319	CCL5,FOS,JUN
	Granzyme A Signaling	3.52	0.105	GZMA,PRF1
	Tumoricidal Function of Hepatic Natural Killer Cells	3.31	0.0833	GZMB,PRF1
	Neuroprotective Role of THOP1 in Alzheimer's Disease	3.27	0.0256	GZMA,GZMB,GZMK
	Renin-Angiotensin Signaling	3.26	0.0254	CCL5,FOS,JUN
	TNFR2 Signaling	3.12	0.0667	FOS,JUN
	Cytotoxic T Lymphocyte-mediated Apoptosis of Target Cells	3.01	0.0588	GZMB,PRF1
T cell subcluster 5	Aldosterone Signaling in Epithelial Cells	14.2	0.0759	DNAJA1,DNAJB1,HSP90AA1,HSP90AB1,HSPA1A/HSPA1B,HSPA5,HSPA6,HSPA8,HSPB1,HSPD1,HSPE1,HSPH1
	Protein Ubiquitination Pathway	14.2	0.0513	DNAJA1,DNAJB1,HSP90AA1,HSP90AB1,HSPA1A/HSPA1B,HSPA5,HSPA6,HSPA8,HSPB1,HSPD1,HSPE1,HSPH1,UBB,UBC
	Unfolded protein response	8.2	0.107	DDIT3,HSPA1A/HSPA1B,HSPA5,HSPA6,HSPA8,HSPH1
	Glucocorticoid Receptor Signaling	7.91	0.0298	ANXA1,CCL5,HSP90AA1,HSP90AB1,HSPA1A/HSPA1B,HSPA5,HSPA6,HSPA8,JUN,TAF7
	Role of PKR in Interferon Induction and Antiviral Response	7.71	0.0598	HSP90AA1,HSP90AB1,HSPA1A/HSPA1B,HSPA5,HSPA6,HSPA8,JUN
	BAG2 Signaling Pathway	7.09	0.116	HSP90AA1,HSPA1A/HSPA1B,HSPA5,HSPA6,HSPA8
	Huntington's Disease Signaling	6.82	0.0338	DNAJB1,HSPA1A/HSPA1B,HSPA5,HSPA6,HSPA8,JUN,UBB,UBC
	eNOS Signaling	5.49	0.0377	HSP90AA1,HSP90AB1,HSPA1A/HSPA1B,HSPA5,HSPA6,HSPA8
	NRF2-mediated Oxidative Stress Response	5.06	0.0317	DNAJA1,DNAJB1,FTL,JUN,SQSTM1,UBB
	Natural Killer Cell Signaling	3.83	0.0254	FYN,HSPA1A/HSPA1B,HSPA5,HSPA6,HSPA8
T cell subcluster 6	Role of IL-17A in Psoriasis	7.76	0.308	CXCL3,CXCL8,S100A8,S100A9
	TREM1 Signaling	7.72	0.08	CCL2,CCL7,CD83,CXCL8,IL1B,TYROBP
	Hepatic Fibrosis Signaling Pathway	6.86	0.0245	CCL2,CXCL8,FOS,FTH1,HIF1A,IL1B,MYL6,SOD2,TIMP1

	Agranulocyte Adhesion and Diapedesis	6.56	0.0363	CCL2,CCL7,CXCL3,CXCL8,IL1B,MYL6,PPBP
	Role of IL-17F in Allergic Inflammatory Airway Diseases	5.59	0.0952	CCL2,CCL7,CXCL8,IL1B
	Granulocyte Adhesion and Diapedesis	5.46	0.0333	CCL2,CCL7,CXCL3,CXCL8,IL1B,PPBP
	Neuroinflammation Signaling Pathway	5.28	0.0233	CCL2,CXCL8,FOS,HLA-DRA,IL1B,SOD2,TYROBP
	Role of IL-17A in Arthritis	5.12	0.0727	CCL2,CCL7,CXCL3,CXCL8
	LXR/RXR Activation	5.08	0.0413	CCL2,CCL7,IL1B,LYZ,S100A8
	Osteoarthritis Pathway	5.07	0.0284	CXCL8,HIF1A,IL1B,NAMPT,S100A8,S100A9
T cell subcluster 7	Oxidative Phosphorylation	6.1	0.0642	MT-ATP6,MT-CO2,MT-CO3,MT-CYB,MT-ND2,MT-ND3,MT-ND5
	Mitochondrial Dysfunction	5.85	0.0468	CASP8,MT-ATP6,MT-CO2,MT-CO3,MT-CYB,MT-ND2,MT-ND3,MT-ND5
	DNA Double-Strand Break Repair by Homologous Recombination	2.72	0.143	ATM,ATRX
	DNA Methylation and Transcriptional Repression Signaling	1.94	0.0571	ARID4B,CHD4
	Sirtuin Signaling Pathway	1.93	0.0172	MT-ATP6,MT-CYB,MT-ND2,MT-ND3,MT-ND5
	Cell Cycle: G2/M DNA Damage Checkpoint Regulation	1.66	0.0408	ATM,MDM4
	Transcriptional Regulatory Network in Embryonic Stem Cells	1.58	0.037	KAT6A,RIF1
	PCP pathway	1.49	0.0333	JUND,ROCK1
	HOTAIR Regulatory Pathway	1.41	0.0187	KMT2A,KMT2C,ROCK1
	Ephrin B Signaling	1.35	0.0278	ITSN2,ROCK1
T cell subcluster 8	Role of IL-17A in Arthritis	3.36	0.0545	CCL5,CXCL8,MAPKAPK2
	Autophagy	3.23	0.0492	CTSL,RB1CC1,SQSTM1
	IL-17A Signaling in Gastric Cells	2.69	0.08	CCL5,CXCL8
	p53 Signaling	2.63	0.0306	HIF1A,JMY,PMAIP1
	Hepatic Fibrosis Signaling Pathway	2.51	0.0136	CCL5,CXCL8,HIF1A,PDCD4,SPP1
	Adipogenesis pathway	2.25	0.0224	DDIT3,HIF1A,NR1D2
	Role of Hypercytokinemia/hyperchemokinemia in the Pathogenesis of Influenza	2.22	0.0465	CCL5,CXCL8
	BAG2 Signaling Pathway	2.22	0.0465	HSPA4,MAPKAPK2
	Unfolded protein response	2	0.0357	DDIT3,HSPA4
	Granulocyte Adhesion and Diapedesis	1.9	0.0167	CCL5,CXCL8,PPBP
T cell subcluster 9	Cdc42 Signaling	20.3	0.0958	ARPC1B,ARPC2,ARPC3,ARPC5,CD3D,CD3G,CFL1,HLA-DPA1,HLA-DPB1,HLA-DQA1,HLA-DQA2,HLA-DRA,HLA-

				DRB1,HLA-DRB5,ITGB1,MYL6
	Actin Cytoskeleton Signaling	13.7	0.0596	ACTB,ACTG1,ARPC1B,ARPC2,ARPC3,ARPC5,CFL1,FLNA,ITGB1,MYL6,PFN1,TMSB10/TMSB4X,TTN
	Antigen Presentation Pathway	13	0.205	CD74,HLA-DPA1,HLA-DPB1,HLA-DQA1,HLA-DQA2,HLA-DRA,HLA-DRB1,HLA-DRB5
	CD28 Signaling in T Helper Cells	12.1	0.0833	ARPC1B,ARPC2,ARPC3,ARPC5,CD3D,CD3G,HLA-DQA1,HLA-DRA,HLA-DRB1,HLA-DRB5
	RhoA Signaling	12	0.0813	ACTB,ACTG1,ARPC1B,ARPC2,ARPC3,ARPC5,CFL1,MYL6,PFN1,TTN
	OX40 Signaling Pathway	11.6	0.1	CD3D,CD3G,HLA-DPA1,HLA-DPB1,HLA-DQA1,HLA-DQA2,HLA-DRA,HLA-DRB1,HLA-DRB5
	Th2 Pathway	11.5	0.0735	CD3D,CD3G,HLA-DPA1,HLA-DPB1,HLA-DQA1,HLA-DQA2,HLA-DRA,HLA-DRB1,HLA-DRB5,MAF
	Regulation of Actin-based Motility by Rho	11.5	0.0957	ACTB,ARPC1B,ARPC2,ARPC3,ARPC5,CFL1,ITGB1,MYL6,PFN1
	Th1 and Th2 Activation Pathway	10.5	0.0585	CD3D,CD3G,HLA-DPA1,HLA-DPB1,HLA-DQA1,HLA-DQA2,HLA-DRA,HLA-DRB1,HLA-DRB5,MAF
	Th1 Pathway	10.5	0.0744	CD3D,CD3G,HLA-DPA1,HLA-DPB1,HLA-DQA1,HLA-DQA2,HLA-DRA,HLA-DRB1,HLA-DRB5
T cell subcluster 10	Oxidative Phosphorylation	31.8	0.303	ATP5F1A,ATP5F1B,ATP5F1C,ATP5F1E,ATP5MC1,ATP5MC3,ATP5MF,ATP5MG,ATP5PB,ATP5PF,ATP5PO,COX5A,COX6A1,COX6B1,COX6C,COX7B,COX8A,NDUFA1,NDUFA11,NDUFA12,NDUFA13,NDUFA2,NDUFA4,NDUFA6,NDUFB1,NDUFB11,NDUFB2,NDUFB3,NDUFB6,NDUFB7,NDUFB9,UQCR10,UQCRQ
	Mitochondrial Dysfunction	31	0.222	ATP5F1A,ATP5F1B,ATP5F1C,ATP5F1E,ATP5MC1,ATP5MC3,ATP5MF,ATP5MG,ATP5PB,ATP5PF,ATP5PO,COX5A,COX6A1,COX6B1,COX6C,COX7B,COX8A,NDUFA1,NDUFA11,NDUFA12,NDUFA13,NDUFA2,NDUFA4,NDUFA6,NDUFB1,NDUFB11,NDUFB2,NDUFB3,NDUFB6,NDUFB7,NDUFB9,PARK7,PRDX3,UCP2,UQCR10,UQCRQ,VDAC1,VDAC3
	Sirtuin Signaling Pathway	21.2	0.127	ATP5F1A,ATP5F1B,ATP5F1C,ATP5F1E,ATP5MC1,ATP5PB,ATP5PF,H1-2,H1-3,H1-4,H1-5,IDH2,LDHB,NDUFA1,NDUFA11,NDUFA12,NDUFA13,NDUFA2,NDUFA4,NDUFA6,NDUFB1,NDUFB11,NDUFB2,NDUFB3,NDUFB6,NDUFB7,NDUFB9,PARP1,PRKDC,RBBP8,SLC25A5,TUBA1B,UCP2,VDAC1,VDAC3,XRCC5,XRCC6
	Granzyme A Signaling	12.9	0.526	ANP32A,GZMA,H1-2,H1-3,H1-4,H1-5,HMGB2,NME1,PRF1,SET
	Actin Cytoskeleton Signaling	10.7	0.101	ACTB,ACTG1,ACTR2,ACTR3,ARPC1B,ARPC2,ARPC3,ARPC4,ARPC5,CDC42,CFL1,FLNA,IQGAP1,MSN,MYH9,MYL6,PFN1,RAC2,RHOA,TLN1,TMSB10/TMSB4X,WAS
	Regulation of Actin-based Motility by Rho	10.3	0.16	ACTB,ACTR2,ACTR3,ARPC1B,ARPC2,ARPC3,ARPC4,ARPC5,CDC42,CFL1,MYL6,PFN1,RAC2,RHOA,WAS
	Remodeling of Epithelial Adherens Junctions	10.1	0.191	ACTB,ACTG1,ACTR2,ACTR3,ARPC1B,ARPC2,ARPC3,ARPC4,ARPC5,IQGAP1,NME1,TUBA1B,TUBB
	Antigen Presentation Pathway	9.21	0.256	CALR,CD74,HLA-DPA1,HLA-DPB1,HLA-DRA,HLA-DRB1,HLA-DRB5,IFNG,PSMB8,PSMB9
	Epithelial Adherens Junction Signaling	9.13	0.112	ACTB,ACTG1,ACTR2,ACTR3,ARPC1B,ARPC2,ARPC3,ARPC4,ARPC5,CDC42,IQGAP1,MYH9,MYL6,RHOA,TUBA1B,TUBB,WAS

	Signaling by Rho Family GTPases	9	0.0861	ACTB,ACTG1,ACTR2,ACTR3,ARPC1B,ARPC2,ARPC3,ARPC4,ARPC5,CDC42,CFL1,GNAI2,IQGAP1,MSN,MYL6,RAC2,RHOA,SEPTIN6,STMN1,VIM,WAS
T cell subcluster 11	Role of IL-17A in Psoriasis	4.96	0.231	CXCL8,S100A8,S100A9
	Polyamine Regulation in Colon Cancer	2.59	0.0909	MXD1,SAT1
	Lipid Antigen Presentation by CD1	2.44	0.0769	FCER1G,PSAP
	Dendritic Cell Maturation	2.44	0.0219	FCER1G,HLA-DRA,PLCE1,TYROBP
	Communication between Innate and Adaptive Immune Cells	2.35	0.0312	CXCL8,FCER1G,HLA-DRA
	Osteoarthritis Pathway	2.22	0.019	CXCL8,FGFR1,S100A8,S100A9
	Cardiac Hypertrophy Signaling (Enhanced)	2.18	0.0123	CACNA1A,CXCL8,CYBB,FGFR1,GNAQ,PLCE1
	GPCR-Mediated Nutrient Sensing in Enteroendocrine Cells	2.17	0.0268	CACNA1A,GNAQ,PLCE1
	LXR/RXR Activation	2.08	0.0248	HMGCR,LYZ,S100A8
	Phagosome Formation	2.04	0.024	CLEC7A,FCER1G,PLCE1

Table S10. Human monocyte gene sets used for module scoring.

Gene Set Name	Description	Reference
Szabo gene sets	Generated from resting and stimulated human T cells isolated from blood and tissues (lungs, lymph nodes, bone marrow).	Szabo et al.(8)
Miao gene sets	Generated from summary of marker genes across the literature (Table S1).	Miao et al.(9)
Cano-Gamez gene sets	Generated from unstimulated and stimulated human T cells, identified by scRNA-seq.	Cano-Gamez et al.(10)

References

1. Villani A-C et al. Single-cell RNA-seq reveals new types of human blood dendritic cells, monocytes, and progenitors. *Science* 2017;356(6335):eaah4573–14.

2. Shay T, Kang J. Immunological Genome Project and systems immunology. *Trends in Immunology* 2013;34(12):602–609.

3. Poel M et al. Transcriptional profiling of human microglia reveals greyâ€“white matter heterogeneity and multiple sclerosis-associated changes. *Nature Communications* 2019;:1–13.

4. The Immunological Genome Consortium et al. Gene-expression profiles and transcriptional regulatory pathways that underlie the identity and diversity of mouse tissue macrophages. *Nat Immunol* 2012;13(11):1118–1128.

5. Gosselin D et al. Environment Drives Selection and Function of Enhancers Controlling Tissue-Specific Macrophage Identities. *Cell* 2014;159(6):1327–1340.

6. Van Hove H et al. A single-cell atlas of mouse brain macrophages reveals unique transcriptional identities shaped by ontogeny and tissue environment. *Nature Neuroscience* 2019;:1–24.

7. Goldmann T et al. Origin, fate and dynamics of macrophages at central nervous system interfaces. *Nat Immunol* 2016;17(7):797–805.

8. Szabo PA et al. Single-cell transcriptomics of human T cells reveals tissue and activation signatures in health and disease. *Nature Communications* 2019;:1–16.

9. Miao YR et al. ImmuCellAI: A Unique Method for Comprehensive T-Cell Subsets Abundance Prediction and its Application in Cancer Immunotherapy. *Adv. Sci.* 2020;7(7):1902880–10.

10. Cano-Gamez E et al. Single-cell transcriptomics identifies an effectorness gradient shaping the response of CD4+ T cells to cytokines. *Nature Communications* 2020;:1–15.
